# Impact of Fermentation of Pumpkin Leaves and Melon Varieties with Lactobacillus Strains on Physicochemical Properties, Antioxidant Activity, and Carotenoid Compounds

**DOI:** 10.3390/foods13223562

**Published:** 2024-11-07

**Authors:** Pretty Mhlanga, Sephora Mutombo Mianda, Dharini Sivakumar

**Affiliations:** 1 Department of Crop Sciences, Tshwane University of Technology, Pretoria 0001, South Africa; prettymhlanga99@gmail.com (P.M.); miandamutombos@tut.ac.za (S.M.M.); 2 Centre for Nutrition & Food Sciences, Queensland Alliance for Agriculture and Food Innovation, The University of Queensland, Brisbane, QLD 4108, Australia

**Keywords:** fermentation, underutilized leafy vegetables, Cucurbitaceae, lactic acid bacteria, functional compounds, sensory properties

## Abstract

This study examined the impact of fermentation using *Lactiplantibacillus plantarum* (*L75*) and *Bifidobacterium longum* (*BF*) on the total soluble solids (TSS), pH, TA, LAB survival, color properties, ascorbic acid content, total phenolic content (TPC), carotenoid components, and antioxidant properties of smoothies made from melon varieties (Cantaloupe, Honeydew, and Watermelon) separately with pumpkin leaves (*Cucurbita moschata* and *Cucurbita pepo*). For all smoothies, pH (r = −0.74) and TSS (r = −0.79) were inversely and strongly correlated with LAB counts, while LAB counts were positively correlated with TA (r = 0.87). Fermentation time (24 to 72 h) significantly (*p* < 0.05) decreased the TSS (%), pH, and color properties of all smoothies fermented with *L75* or *BF*, while TA increased. Fermenting Cantaloupe melon and *C pepo* leaves with *L75* (CMCL75) for 24 h increased the ascorbic acid content to 3.8 mg/100 mL. The sensory panel scores were highest for Watermelon and *C. moschata* or C. pepo fermented with L75 or BF for 24 h. TPC concentration was highest in CMCL75 (70.76 mg of gallic acid per 100 mL) after 24 h. *C. pepo* leaves and Cantaloupe fermented with *L75* (CPCL75) showed the highest concentration of total carotenoids (70.38 mg/100 mL), lutein (2.53 µg/100 mL), cis β-carotene (25.43 µg/100 mL), and trans β-carotene (620.37 µg/100 mL). In contrast, CMCL75 showed the highest concentration of zeaxanthin (0.70 mg/100 mL). This study demonstrated the potential of fermenting Cantaloupe and pumpkin leaves together with the *L75* strain to produce non-dairy functional products.

## 1. Introduction

Melons belong to the Cucurbitaceae family, and the estimated global consumption is around 4.25 kg per person. The World Health Organization recommends consuming more than 400 g of fruits and vegetables daily to improve overall health and reduce the risk of various non-communicable diseases [1]. Melons provide several health benefits thanks to antioxidants like lycopene and vitamin C. Lycopene has been linked to the prevention of numerous chronic conditions, including cancer and diabetes [2]. Carotenoids, such as β-carotene found in Cantaloupe melons, are converted by the body into vitamin A (retinol). Lycopene primarily exists in its all-E-isomer configuration in foods. During processing, lycopene can undergo Z and E isomerization due to oxidation and heat. Reports indicate that only the E-isomers of 5Z-lycopene are biologically active. Given its polyene structure and electron-rich properties, lycopene is suitable for electrophilic reactions, able to scavenge free radicals, and is highly reactive [3]. Additionally, Honeydew melons are a good source of vitamin B (thiamine), potassium, and vitamin C. They are low in calories, containing only 36 calories per 100 g [4]. Watermelon is one of Africa’s 101 most important orphan crops. Considering the nutrient-dense benefits of orphan or underutilized crops and their integration into diverse food systems, orphan crops have recently become a topic of interest. Since ancient times, pumpkin leaves have been eaten in Africa. In addition to being nutritious and functional, pumpkin leaves contain phenolics, flavonoids, and beta-carotene, and are active antioxidants [5]. In a 100 g serving, fresh pumpkin leaves contain 38 mg of magnesium, 39 mg of calcium, 15.90 mg of iron, 3.15 g of protein, and no cholesterol [6]. Starch-based foods in developing countries are low in micronutrients, so consuming leafy vegetables, such as pumpkin leaves, boosts daily micronutrient intake.

Approximately 40% of Watermelons are lost by producers and marketers, and some lose more than 60% [7]. Moreover, improper packaging materials can facilitate spoilage microorganism proliferation by trapping water released by pumpkin leaves during respiration or transpiration [8]. Post-harvest losses harm the economy, environment, and health. Moreover, the United Nations calls on people to reduce food loss by half by 2030 to improve global food security and the population’s health. In addition, food waste contributes 6% to global carbon emissions [9]. A significant amount of pressure is also being put on small- and medium-scale retailers by the cost of energy and the transition from coal to renewable energy to maintain cold chain infrastructure [10].

The thermal processing of fruit and vegetable juices for microbial protection is a widely used preservation technique. The thermal processing of juices has a detrimental impact on important nutritional compounds, such as vitamins like ascorbic acid, thiamine, and folic acid [11]. Juice extraction, however, eliminates insoluble dietary fibers, despite their health benefits [12]. In this way, smoothies provide consumers with the nutritional benefits of fruits and vegetables. A smoothie is a semi-liquid nutrient-dense product consisting of fruit and vegetables [13]. 

Furthermore, lactic acid fermentation is an effective strategy for reducing post-harvest losses and preserving nutrients. Dairy probiotics may contain allergens, and a significant portion of carbon dioxide emissions from food production comes from animal products [14], prompting the food industry to explore alternative microbial delivery systems. This fermentation process helps preserve food, extend shelf life, enhance nutritional value, and improve flavor and texture. Research by Moloto et al. [15] shows it can also recover bioactive compounds from underutilized vegetables. Various melons can be used as dietary foods or natural flavorings. The global food and beverage market is projected to reach USD 7464.2 billion from 2022 to 2027 [16]. 

The use of lactic acid bacterium (LAB) cultures enhances the biological value of fermented foods. Additionally, incorporating indigenous fruits or vegetables into smoothies can improve their nutritional content. LAB fermentation boosts antioxidant activity by releasing bioactive compounds through the metabolism and biotransformation of phenolic compounds. *Lactiplantibacillus plantarum* (*L. plantarum*) is commonly added to fermented foods to enhance their quality and health benefits [17]. Meanwhile, *Bifidobacterium longum* (*B. longum*), a non-pathogenic, Gram-positive, catalase-negative, rod-shaped bacterium, is naturally found in the gastrointestinal tract of humans and is frequently added to various food products [18]. This study aims to reduce food waste from melons and pumpkin leaves while rescuing bioactive compounds, particularly carotenoids, through fermentation technology utilizing specific lactobacillus strains for the benefit of consumers. However, limited information is available regarding the changes in bioactive compounds—especially carotenoid components—and antioxidant activity in different types of melons after fermentation with leafy vegetables.

To develop fermented smoothies, it is essential to choose melon varieties based on their physical and sensory characteristics. Therefore, this study investigates the effects of fermentation by two lactobacillus strains—*Lactiplantibacillus plantarum* and *Bifidobacterium longum*—on the physicochemical properties, bioactive compounds (total phenols, total carotenoid content, and specific carotenoid components), antioxidant activities, and sensory properties of smoothies made from pumpkin leaves (*Cucurbita moschata* and *Cucurbita pepo*) and the melon varieties *Citrullus lanatus* and *Cucumis melo*, separately.

## 2. Materials and Methods

### 2.1. Chemicals

This study was conducted with chemicals and reagents supplied by Merck Life Science (Pty) Ltd. (Johannesburg, South Africa).

### 2.2. Creating Smoothies with Plant Material

This study utilized two accessions of pumpkin leaves, namely *Cucurbita moschata* and Cucurbita pepo, alongside three varieties of melons: Watermelon, Cantaloupe, and Honeydew. The pumpkin leaves were sourced from the Tshiombo Cooperative in Venda, located in the Limpopo province of South Africa, while the melons were purchased from a commercial farm within the same province. To prepare the pumpkin leaves, they were cleaned with tap water to remove excess soil. After that, the leaves were immersed in a 0.01% sodium hydroxide solution, rinsed again with sterile water, sliced, and dried. The melons were peeled and diced using stainless steel knives in a sterile environment. Smoothies were prepared by blending and homogenizing 800 g of each type of melon fruit with 200 g of each pumpkin leaf accession (*C. moschata* or *C. pepo*) separately, along with 500 mL of distilled water. This process was carried out in a Russell Hobbs blender (700 W). Following this, the smoothies underwent pasteurization for 30 min in a water bath (WBE28A12E, PolyScience, Niles, IL, USA) at 63 °C. After pasteurization, the smoothies were cooled on ice before being inoculated with lactic acid bacterium (LAB) strains, specifically *L. plantarum* (L75) and *B. longum* (BF).

### 2.3. Smoothie Starter Cultures and Fermentation

Lactobacillus (LAB) strains (*Lactiplantibacillus plantarum* (*L. plantarum*) (*L75*) and *Bifidobacteria longum* (*B. longum*) (*BF*)) were obtained from the Fruit and Vegetable Technology culture collection at Tshwane University of Technology, Pretoria, South Africa. The strains were revived and multiplied by sequential suspension in 9 mL of de Man, Rogosa, and Sharpe (MRS) broth and incubated anaerobically for 48 h at 30 °C, as reported by Nirina et al. [19]. In sterile distilled water, the resulting cells were re-suspended after centrifugation at 12,000× *g* for 5 min at 4 °C. Spectrophotometer (UV-1750 UV-Vis spectrometer, Shimadzu, Suzhou, China) was used to determine culture concentrations (turbidity method). The pre-culture concentration was calibrated to 0.05 McFarland standard (1–5 × 10^8^ CFU/mL) at a 660 nm wavelength. Subsequently, a 1% inoculum was introduced into smoothies, as outlined by Managa et al. [20]. The smoothies were inoculated with each strain separately. The smoothies were incubated (fermented) at 37 °C for 2 h, 24 h, 48 h, and 72 h. Fermented smoothies were stored at −20 °C until analysis after fermentation. The non-inoculated smoothies served as the control. The fermentation was performed in triplicate.

### 2.4. Survival of LABs

At different fermentation time intervals, the viability of the LAB strains and the safety of the smoothies were assessed. Pour plate counts were conducted, as described by Managa et al. [20], to enumerate the colonies on medium plates. Concisely, 100 µL of smoothie was fused with 900 µL of sterile distilled water and then further diluted to a factor of 10^−6^. Concisely, 100 µL of the diluted sample was placed on suitable plate media, i.e., MRS agar, nutrient agar (NA), xylose lysine deoxycholate agar (XLD), and Eosin-methylene blue agar (EMB) to measure the growth of surviving LABs, aerobic bacteria, *Salmonella*, and *E. coli*, respectively. Plated Petri dishes were incubated at 37 °C for 48 h for MRS, and 24 h for NA, XLD, and EMB. Cell viability was expressed in CFU/mL of smoothie, as described by Managa et al. [20]. 

### 2.5. Assessment of Physicochemical Parameters

The pH of both inoculated and non-inoculated treatments was evaluated using a digitally calibrated pH meter (Melter-Toledo Instruments Co., Shanghai, China), as outlined by Managa et al. [20]. Total acidity (TA) measurements were taken using 0.1 N NaOH and the phenolphthalein titration method [20]. Total soluble solids (TSSs) were assessed with a pocket refractometer (Agato pocket PAL-2, Tokyo, Japan). Distilled water was used to calibrate the refractometer before each test to enhance the accuracy of the results. Color values for all treatments were recorded using a colorimeter (CR-400 Chroma Meter, Konica Minolta Sensing, Shanghai, China), which was calibrated with a white standard tile. The color values were expressed in the CIEL*a*b* color space, where L* indicates lightness or luminosity (0 = dark and 100 = bright), a* represents the amount of green (−) and red (+) color, and b* measures the amount of blue (−) and yellow (+) color. The color change or difference was calculated using the L*a*b* readings and expressed as *ΔΕ* [20]. In this context, L*0, a*0, and b*0 represent the color parameters for fresh melon (Watermelon, Cantaloupe, or Honeydew melon) and pumpkin leaf (*C. moschata* and *C. pepo*) smoothies, while L*, a*, and b* denote the corresponding values after fermentation.

$$\Delta E=\sqrt[{2}]{(L^{*}-L_{0}^{*}{)}^{2}+(a^{*}-a_{0}^{*}{)}^{2}+(b^{*}-b_{0}^{*}}{)}^{2}\;$$

### 2.6. Ascorbic Acid Content

The samples were analyzed for ascorbic acid content using the metaphosphoric acid and 2,6-dichloroindophenol dye titration method, as outlined by Mashitoa et al. [21]. Titration was performed using a 10 mL aliquot of the solution against the dye solution, with a pink color indicating the titration endpoint. The ascorbic acid concentration was expressed in milligrams per 100 g on a fresh weight basis. To calculate the ascorbic acid content, Equation (1) was utilized.

Ascorbic acid (mg/100 g) = Titre × Dye factor × volume made × 100(1)

(Aliquot of extract taken × Weight of sample)

### 2.7. Total Phenolic Content (TPC)

The Folin–Ciocalteu (F-C) assay [22] was employed to determine the total phenolic content (TPC) in smoothies. For each treatment, 2 mL of smoothie was collected and homogenized with 2 mL of 80% methanol. The mixture was vortexed and sonicated for 10 min at 30 °C. In conical-bottom Eppendorf tubes (LABOCARE™, Strathavon 2031, Johannesburgh, South Africa), 100 µL of the extracted samples were combined with 750 µL of F-C reagent and allowed to react for 5 min. Following this, sodium carbonate (Na_2_CO_3_) was added to the mixture, which was then incubated for 2 h. The samples were plated onto 96-well microplates (BMG LABTECH GmbH, Spectro Star Nano, Ortenberg, Germany), and the absorbance was measured at 750 nm using a spectrophotometer (UV-1750 UV-Vis spectrometer, Shimadzu, Suzhou, China). The concentration of TPC was calculated using the calibration curve equation Y = 0.0019x − 0.1733, with an R^2^ value of 0.8511, employing 1 µM gallic acid as the standard.

### 2.8. Total Carotenoid Content and Carotenoid Profile

As outlined by Djuikwo et al. [23], the smoothies were analyzed for total carotenoid content (TCC) and individual carotenoids. A 2 mL sample of each smoothie was mixed with 5 mL of acetone and 5 mL of 95% ethanol containing 0.1% butylated hydroxytoluene (BHT). The mixture was vortexed and sonicated for 10 min at 30 °C using a vortex mixer (Benchmark Scientific Inc., Taiwan) and a DC-150H MRC ultrasonic cleaner. After the initial sonication, 10 mL of 20% potassium hydroxide (KOH) was added to the solution, which was then sonicated for an additional 10 min. To separate the carotenoids, 10 mL of a hexane/dichloromethane solution (70:30 *v*/*v*) containing 0.1% BHT was added and sonicated for 5 min at 30 °C. Following this, 5 mL of sodium chloride (NaCl) solution was added, and the mixture was centrifuged at 3900× *g* for 5 min. Under a nitrogen stream, the extracted samples were collected three times. To reconstitute the fresh extracts for profiling and total carotenoid content analysis, 2 mL of a methanol/MTBE solution (50:50 *v*/*v*) was added. An aliquot of 200 µL was then placed in 96-well microplates (BMG LABTECH GmbH, Spectro Star Nano, Ortenberg, Germany) for total carotenoid content analysis, with the data expressed in mg per 100 mL for TCC.

The HPLC (Shimadzu, LC-2030C 3D, Tokyo, Japan) analysis for the quantification of individual carotenoids was conducted as described by Bhengu et al. [24] without any modification. An aliquot of 1000 µL was withdrawn from the reconstituted samples and transferred into HPLC vials. The separation of compounds was performed on an InertSustain C30 column (3.0 × 250 mm, 3 µm, GL Sciences, Tokyo, Japan) using a gradient elution of 0.1% of formic acid in methanol (solvent A) and methyl tert-butyl ether (solvent B) at a flow rate of 0.3 mL/min. Chromato-grams were read at 460 nm. The quantification of carotenoids was achieved using calibration curves constructed with lutein, β-carotene, lycopene, and zeaxanthin, and is expressed in mg/100 mL. 

### 2.9. Antioxidant Activity

#### 2.9.1. Ferric-Reducing Antioxidant Power (FRAP)

The FRAP assay was performed as described by Benzie and Strain [25] with some modifications outlined by Llorachet al. [26]. The working solution of FRAP was prepared by mixing 25 mL of acetate buffer with 2.5 mL of TPZ (2,4,6-tripyridyl-*S*-triazine) solution and 2.5 mL of ferric achloride. The mixture was shaken at 37 °C using a water bath (WBE28A12E, PolyScience, Niles, IL 60714-4516, USA). For the final FRAP analysis, 75 µL of extracted samples were mixed with 1425 µL of FRAP working solution and incubated for 30 min in the dark at room temperature. After the incubation time lapsed, 200 µL of each sample treatment was plated on the 96-well microplates (BMG LABTECH GmbH, Spectro Star Nano, Ortenberg, Germany) and the absorbance was measured at 593 nm on a spectrophotometer (UV-1750 UV-Vis spectrometer, Shimadzu, Suzhou, China). Trolox was used as the standard for FRAP and the results were expressed as mM TEAC/mL on a fresh weight basis.

#### 2.9.2. 1,1-Diphenyl-2-Picrylhydrazyl (DPPH)

The capability of the fermented extracts to scavenge the radicals of DPPH was determined utilizing the spectrophotometric methods [15]. To prepare the DPPH solution, 6 mg of DPPH was dissolved in 25 mL of solvent (80% methanol), while a working solution was prepared by homogenizing 10 mL of DPPH solution with 50 mL of solvent. DPPH's scavenging ability was further determined by plating different concentrations of the extracted samples (10–100 µL), solvent (90–0 µL), and constant concentration (200 µL) of DPPH on the 96-well microplates (BMG LABTECH GmbH, Spectro Star Nano, Ortenberg, Germany). The plated solutions were incubated for 10 min in the dark; thereafter, the absorbance of the mixture was read at 593 nm utilizing a spectrophotometer (UV-1750 UV-Vis spectrometer, Shimadzu, Suzhou, China). The solvent was used as a blank. The scavenging activity % RSA of the samples was calculated using the formula below, and the results were expressed as IC_50 µ_L/mL of pumpkin leaf–melon smoothies on a fresh weight basis:

Scavenging (%) = [A_a_ − (A_b_ − A_c_)]/A_a_ × 100

where A_a_ is the absorbance of the DPPH solution without adding the solvent; A_b_ is the absorbance of the mixture solution containing both the sample and DPPH; and A_c_ is the absorbance of the blank solution with DPPH.

#### 2.9.3. 2,2-Azinobis-(3-Ethyl-Benzothiazoline-6-Sulfonic Acid) (ABTS)

The ABTS^+^ cation radical was created by combining 5 mL of ABTS solution with 145 mL of phosphate buffer solution, which was left to incubate for 12 to 16 h in the dark at 25 °C [15]. Before ABTS analysis, a precise 100 µL of the sample extract was diluted into 900 µL of solvent. Following samples dilution, different concentrations of the samples (10–100 µL), solvent (90–0 µL), and uniform concentration (200 µL) of ABTS^+^ cation radical were plated on 96-well microplates and read on a spectrophotometer at an absorbance of 593 nm. Like DPPH, the solvent was used as a blank and the scavenging activity % RSA of the samples was calculated using the formula described in Section 2.9.2. The results of ABTS were expressed as the IC_50_ µL/mL of pumpkin leaf–melon smoothies on a fresh weight basis.

### 2.10. Sensory Analysis

The panel consisted of 40 untrained individuals, both males and females between the ages of 18 and 53. The panelists were all familiar with the taste of pumpkin leaves and melons. Before the session, the panel was briefed on how smoothies are made. Each panelist received a coded smoothie from the different fermentation hours in shot glasses, still water, sensory forms, and pens. Additionally, the samples were presented in a completely random order throughout the session. The smoothies were tested for color, taste, aroma, and overall acceptability. Following each test, water was used to clean the mouth. Using a scale of 0–9 with 0 being extremely disliked, 5 being neither like nor dislike, and 9 being extremely like, intensity scores were assigned based on individual panelists’ degree of liking a product.

### 2.11. Statistical Analysis

This experiment utilized a completely randomized design, featuring three replicates for each treatment, and was conducted twice. The statistical analysis was performed using GenStat 64-bit release 22.1 (Hempstead, England). A two-way analysis of variance (ANOVA) was conducted for the survival of lactic acid bacteria (LABs), total soluble sugars, pH and titratable acidity (TA), color properties, and ascorbic acid levels. In contrast, total phenolics, carotenoid content, carotenoid components, and antioxidant scavenging activities were analyzed using one-way ANOVA. Fisher’s protected least significant difference (LSD) test was employed for all analyses to determine significant differences at a threshold of *p* < 0.05. Additionally, mean scores from sensory analysis were calculated, and significant variations were assessed using ANOVA, followed by Fisher’s LSD test, to identify differences at *p* < 0.05.

## 3. Results and Discussion

### 3.1. LAB Survival

**Figure 1:**
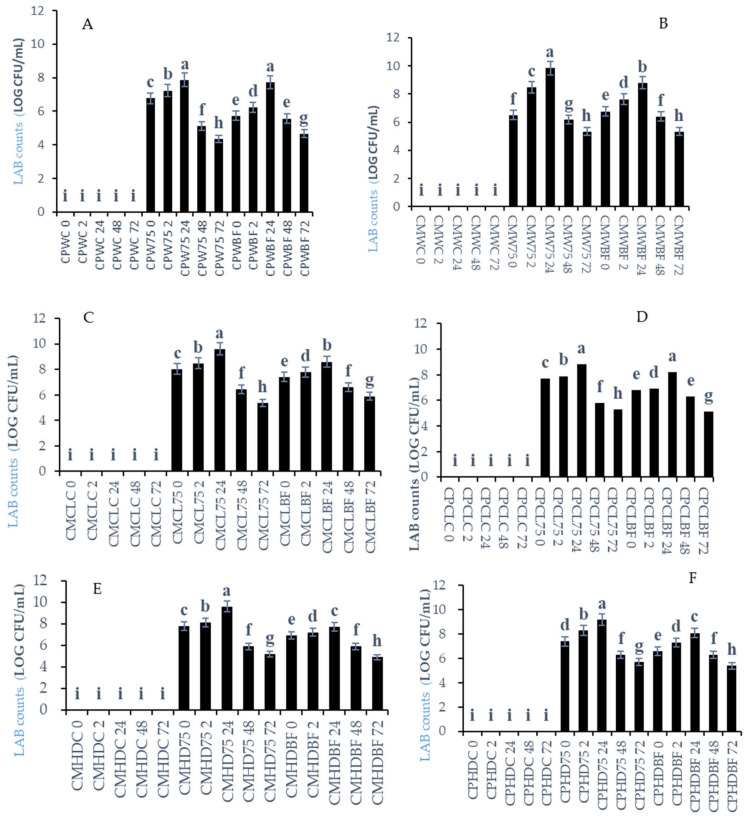
The population of *Lactiplantibacillus plantarum* (*L75*) and *Bifidobacterium longum* (*BF*) in smoothies of different melon types and pumpkin leaves (*Cucurbita moschata* and *Cucurbita pepo*) after 2, 24, 48, and 72 h of fermentation. Mean values were calculated based on three replicate samples. Different letters in the same bar refer to statistical difference at *p*  <  0.05 according to Fisher’s protected LSD test. (**A**) *Cucurbita pepo* + Watermelon smoothies fermented with LABs; (**B**) *Cucurbita moschata* + Watermelon smoothies fermented with LABs; (**C**) *Cucurbita moschata* + Cantaloupe smoothies fermented with LABs; (**D**) *Cucurbita pepo* + Cantaloupe smoothies fermented with LABs; (**E**) *Cucurbita moschata* + Honeydew melon smoothies fermented with LABs; (**F**) *Cucurbita pepo* + Honeydew melon smoothies fermented with LABs. *Keys: Cucurbita moschata* + Watermelon (CMWC), *Cucurbita moschata* + Watermelon + *L75* (CMW75), *Cucurbita moschata* + Watermelon + *B. longum* (CMWBF), *Cucurbita pepo* + Watermelon Control (CPWC), *Cucurbita pepo* + Watermelon + *L75* (CPW75), *Cucurbita pepo* + Watermelon + *B. longum* (CPWBF), *Cucurbita moschata* + Cantaloupe Control (CMCLC), *Cucurbita moschata* + Cantaloupe + *L75* (CMCL75), *Cucurbita moschata* + Cantaloupe + *L75* (CMCL75), *Cucurbita pepo* + Cantaloupe Control (CPCLC), *Cucurbita pepo* + Cantaloupe + *L75* (CPCL75), *Cucurbita pepo* + Cantaloupe + *B. longum* (CPCLBF), *Cucurbita moschata* + Honeydew Control (CMHDC), *Cucurbita moschata* + Honeydew + *L75* (CMHD75), Cucurbita moschata + Honeydew + *B. longum* (CMHDBF), *Cucurbita pepo* + Honeydew Control (CPHDC), *Cucurbita pepo* + Honeydew + *L75* (CMHD75), and *Cucurbita pepo* + Honeydew + *B. longum* (CPHDBF).

According to Figure 1, LAB activity was not present in all the controls, as they were not inoculated with the fermentation cultures. The different melon and pumpkin leaf smoothies were inoculated with *L75* or *B. Longum* at 6 log cfu /mL. However, the population of the inoculated smoothies ranged between log 4.3 and log 9.8 cfu/mL of LABs during the fermentation process and further peaked at 24 h of fermentation. According to our findings, LAB survival was also affected by increased TA (lactic acid) when fermentation time was increased from 48 to 72 h. Overall, smoothies inoculated with *L75* had the highest colony counts. *L75* populations in different melon and pumpkin leaf smoothies after 24 h were shown as fold increase compared to the inoculated LAB counts as follows: in *C. moschata* + Watermelon + *L75*(CMW75)-1.27-fold increase, *C. moschata* + Cantaloupe + *L75* (CMCL75)-1.26-fold increase, *C. moschata* + Honeydew + *L75* (CMHD75)-1.26-fold increase, *C. pepo* + Honeydew + *L75* (CMHD75)-1.23-fold increase, and *C. pepo*+Honeydew + *B. longum* (CPHDBF)-1.23-fold increase. However, the increase was not significant (*p* < 0.5). On the other hand, although *C. pepo* + Cantaloupe + *L75* (CPCL75), *C. pepo* + Watermelon + *B. longum* (CPWBF), and *C. pepo* + Watermelon combined with *L75* (CPW75) increased lactic acid bacterium (LAB) counts with increases of approximately 1.19-fold, 1.15-fold, and 1.14-fold. However, CPCL75, CPWBF, and CPW75 exhibited significantly lower increases in LAB counts compared to CMW75, CMCL75, CMHD75, and CPHDBF smoothies (*p* < 0.05).

Thus, combinations such as *C. moschata* + Watermelon, *C. moschata*+ Cantaloupe, and *C. pepo* + Honeydew, as well as *C. pepo* + Cantaloupe, demonstrated higher cell counts, exceeding 9.1 log cfu/mL (for *L75* and *B. longum*), than the *C. pepo* + Watermelon smoothies. According to Mandha et al. [27], Lactobacillus strains fermented for 24 h showed an increase from 8 to 9 log cfu/mL during the lactic acid fermentation of Watermelon juice. They reported a significant growth (*p* < 0.05) with *L75*, which reached 9.23 log cfu/mL. Furthermore, Fonteles et al. [28] noted an increase in the viability of *L. casei* to 8.3 log cfu/mL in Cantaloupe juice. This indicates that melons serve as a suitable matrix for the survival of LABs.

According to Mandha et al. [27], LABs fermented for 24 h increased to 8–9 log cfu/mL during the lactic acid fermentation of Watermelon juice. Furthermore, Fonteles et al. [28] reported an increase in *L. casei* viability in Cantaloupe juice cells of 8.3 log cfu/ mL. Therefore, melons are a suitable LAB matrix for the survival of LABs. Moreover, Mashitoa et al. [21] showed that the fermentation with *L75* and leaves of the sweet potatoes of orange-fleshed ‘Bophelo’ and ‘Beauregard’ increased the *L75* survival after 24 h, which ranged from 8 to 10 log cfu/ mL. On the contrary, the leaves of purple-fleshed ‘08.21P’ and cream-fleshed ‘Ndou’ displayed an increase in *L75* to log 8 cfu/ mL and 10 log cfu/mL after 48 h. The differences in survival of *L75* and fermentation hours again depend on the substrate used for fermentation and the build-up of metabolites. Therefore, this study shows that LAB survival is dependent on the type of melons and the variety of pumpkin leaves. For a food to be labelled as a probiotic, it must possess log 6 cfu/gm or mL of probiotic microorganisms [29]. To be effective, fermented probiotic products should contain a high concentration of viable bacterium cells [29]. Also, 100 mL or grams of a product must ideally contain around 7 log^−10^ cfu/ mL viable cells [30]. According to our results, we obtained viable cells at a probiotic dosage of above log 7 cfu/ mL for the three types of melons and pumpkin leaves fermented by *L75* or *B. longum.* The pH dropped as the LAB population increased, indicating a negative correlation (r = −0.74). Contrary to this, the LAB population increased with increasing TA and showed a positive correlation (r = 0.87). However, LAB survival may decrease during fermentation due to the continuous decrease in pH and the increase in acidic environments, since acids hinder bacterial viability and growth by inhibiting enzymatic reactions, leading to acidity in the cytoplasm and requiring more energy to maintain intracellular pH [31]. Furthermore, the decrease in sugars during fermentation may have slowed down the fermentation process. The data collected indicate that no pathogens, fungi, or bacteria grew in any of the plated media, indicating that the smoothies are safe for consumption.

### 3.2. Total Soluble Sugars (TSSs)

The sugar index is often used to measure total soluble solids (TSSs), a quality indicator related to sweetness [32]. At 0 h of fermentation, all three non-inoculated melon type (Cantaloupe, Honeydew melon, and Watermelon) and pumpkin leaf (*C. moschata*/*C. pepo*) smoothies had higher TSSs than their fermented samples (Table 1). There was a significant drop in TSSs (%) with increasing fermentation time (24 to 72 h) for the three types of melon (Cantaloupe, Honeydew, and Watermelon) and pumpkin leaves (*C. moschata*/*C. pepo*) fermented with *L75* or *B. longum*, but non-inoculated smoothies (0 h) of these respective samples showed higher TSS levels. A reduction in TSSs (%) compared to the 0 h control samples was shown for all types of melon and pumpkin leaves with respect to 24, 48, and 72 h in Table 1. The bioconversion of sugar into lactic acid and the consumption of sugar for the growth and metabolism of bacteria can lead to a reduction in TSSs. For example, Managa et al. [20] reported a decrease in TSSs in chayote-pineapple smoothie fermented with LAB strains *L75* and *Weissella cibaria* (*W64*). Additionally, Kaprasob et al. [33] found that *L. plantarum* consumes sugar more rapidly than other bacteria during the fermentation of cashew apple juice. This observation supports the claim of decreased TSSs in *L. plantarum*-fermented mango juice after 72 h [34]. A decrease in TSSs suggests that the culture can exploit sugars in smoothies of melon types for the growth of both LAB strains. In contrast, the fermentation of the three types of melons and pumpkin leaves (*C. moschata/C. pepo*) with *B. longum* for 72 h significantly (*p* < 0.05) decreased the TSSs. This indicates that some probiotic strains were found to be capable of growth in different fruit matrices [34]. Kumar et al. [34] showed that the sugar utilization pattern can be used to determine whether bacteria rapidly consumed the substrate (sugars) in the fruit matrix.

Different strains of *Bifidobacterium longum* have been found to ferment carbohydrates to varying degrees, with the majority being able to ferment fructose, glucose, sucrose, and raffinose [35]. Different types of melon may contain other soluble carbohydrates. However, an increase in TSSs at 2 h of fermentation might be due to the capability of complex carbohydrates present in the introduced fruit and vegetable commodities to biotransform into simple sugar molecules, thus increasing the values of TSSs [33]. Compared to Watermelon and pumpkin leaf smoothies, the following combinations showed TSS levels < 4.0 after fermentation: *C. moschata* + Honeydew + *B. longum* (CMHDBF) and *C. pepo* + Honeydew + *B. longum* (CPHDBF) after 24 h, 48 h, and 72 h of fermentation; *C. moschata* + Honeydew + *L75* (CMHD75) after 24 h, 48 h, and 72 h of fermentation; *C. moschata* + Cantaloupe + *L75* (CMCL75) after 48 h and 72 h of fermentation; *C. moschata* + Cantaloupe + *B. longum* (CMCLBF) after 72 h of fermentation; and *C. pepo* + Cantaloupe + *B. longum* (CPCLBF) after 48 h and 72 h of fermentation. Possibly due to substrate effects (fruit matrix), the nutrient composition influencing fermentation and TSSs decreases [35]. Hou et al. [36] reported that carbohydrate fermentation varied depending on substrate type and even fermentation time. The reduction in sugar content is unappealing to taste. However, the consumption of low-sugar smoothies can help regulate blood sugar levels for individuals with diabetes.

### 3.3. pH and TA

The pH value of lactic acid fermentation plays a crucial role in its physicochemical properties. Fermented smoothies made from different melon types and *C. moschata* or *C. pepo* showed a significant decrease in pH with increasing fermentation time (Table 1). The pH of non-inoculated smoothies was higher than that of inoculated smoothies. The following fermentation treatments showed a pH lower than 4 at 72 h: *C. moschata +* Watermelon *+ L75* (*CMW*75), *C. pepo* + Watermelon + *L75* (CPW75), *C. moschata* + Cantaloupe + *L75* (CMCL75), *C. pepo* + Cantaloupe + *L75* (CPCL75), *C. moschata* + Honeydew + *L75* (CMHD75), *C. moschata* + Watermelon + *B. longum* (CMWBF), *C. moschata* + Cantaloupe + *B. longum* (CMCLBF), *C. pepo* + Cantaloupe + *B. longum* (CPCLBF), *C. moschata* + Honeydew + *B. longum* (CMHDBF), and *C. pepo* + Honeydew + *B. longum* (CPHDBF). The growth of LABs could have contributed to pH decrease, while pH was inversely and strongly correlated to LAB counts in melon types and *C. moschata* or *C. pepo* (r = −0.74, *p* < 0.05) smoothies fermented with *L75* and *B. longum*. The pH values dropped remarkably during the fermentation of chayote leaf (*Sechium edule*) and pineapple with lactic acid bacterium (LAB) strains such as *Lactobacillus plantarum* (*L75*), *Weissella cibaria* (*W64*), and their combination (*LW64* + *75*) from 3.46 to 2.50 [20]. Also, Mandha et al. [37] showed a similar decrease in pH in Watermelon and mango juices fermented by *Levilactobacillus (L.) brevis, Lacticaseibacillus (La.) casei,* and *Pediococcus (P.) pentosaceus.* The continuous decline in pH during fermentation could be attributed to the actions of LAB strains generating organic acids and CO_2_ through the metabolic pathways of carbohydrates with resulting carbonic acids lowering pH during the metabolic process [38]. It is advantageous to reduce the pH level of food during fermentation to stimulate the growth of LABs and lactic acid producers, and to inhibit the growth of pathogenic bacteria *Listeria monocytogenes* and *Escherichia coli* O157:H7, while maintaining 4.6 pH for food safety [39]. In all treatments, TA levels increased with fermentation time, and the highest concentration of TA (Equivalent Lactic Acid g/100 mL) was observed at 72 h for all melon types and pumpkin leaves (*C. moschata*/*C. pepo*). Fresh Watermelon juice contains malic acid (dicarboxylic acid) and, during fermentation, it transforms into lactic acid and CO_2_ [39]. A decline in pH value and TSSs was correlated with an increase in the TA of smoothies of melon types and pumpkin leaves (*C. moschata*/*C. pepo*).

### 3.4. Color Properties

Color is a crucial parameter that influences how consumers perceive and accept fruits and vegetables. Supplementary (S) Table S1 shows the color properties and color change *(*∆*E*) values of smoothies. After 48 and 72 h of fermentation, the ∆*E* increased significantly (*p* < 0.05) in all three melon types and *C. moschata* or *C. pepo* leaves fermented with *L75* or *B. longum*. The color degradation may be due to the breakdown of pigments due to observed pH reductions and acidic conditions (Supplementary Table S1). As part of the CIELab three-dimensional space, there are three components: luminosity (L*  =  0 indicates black, and L*  =  100 implies colorless), red/green (a*  >  0 is in correspondence with red, and a*  <  0 is correlated with green), and the yellow/blue color component (b*  >  0 is in correspondence with yellow, and b*  *<  *0 is linked with blue) [34]. However, when Watermelon, *C. moschata*, or *C. pepo* leaves were fermented with *L75* or *B longum*, the brightness (L*), yellowness (b*), and redness (a*) decreased with increasing fermentation time compared to the unfermented samples (0 h). Conversely, in Cantaloupe, *C. moschata*, or *C. pepo* leaves fermented with *L75* or *B longum*, the brightness (L*) and yellowness (b*) decreased while the redness (a*) decreased with increasing fermentation time compared to the unfermented samples (0 h). Contrary to this, the brightness (L*) and yellowness (b*) decreased when Cantaloupe or Honeydew melons and *C. moschata* or *C. pepo* leaves were fermented with *L75* or *B longum*. Additionally, the redness (a*) decreased with increasing fermentation time compared to the unfermented samples (0 h). 

Mannaga et al. [20] demonstrated that fermenting chayote leaves (Sechium edule) and pineapple with lactic acid bacterium (LAB) strains, specifically L75 and *Weissella cibaria* (W64), as well as their combination (LW64 + L75), impacted the color properties of the leaves. This effect was likely due to enzymatic oxidation during the fermentation process. Rocha and Morais [40] showed that the onset of browning reactions can result in a decrease in lightness and an increased a* (redness) value. Moreover, Watermelon pigments (lycopene and β-carotene) and Cantaloupe (carotenoids) could have been broken down by heat during pasteurization (Section 2.2) [39]. In addition, fermentation may further degrade these carotenoids into aroma volatiles such as monoterpenes, geranyl acetone, and geraniol [39]. The color change in all smoothies was reduced at 24 h of fermentation compared to 48 h and 72 h.

### 3.5. Ascorbic Acid

**Figure 2:**
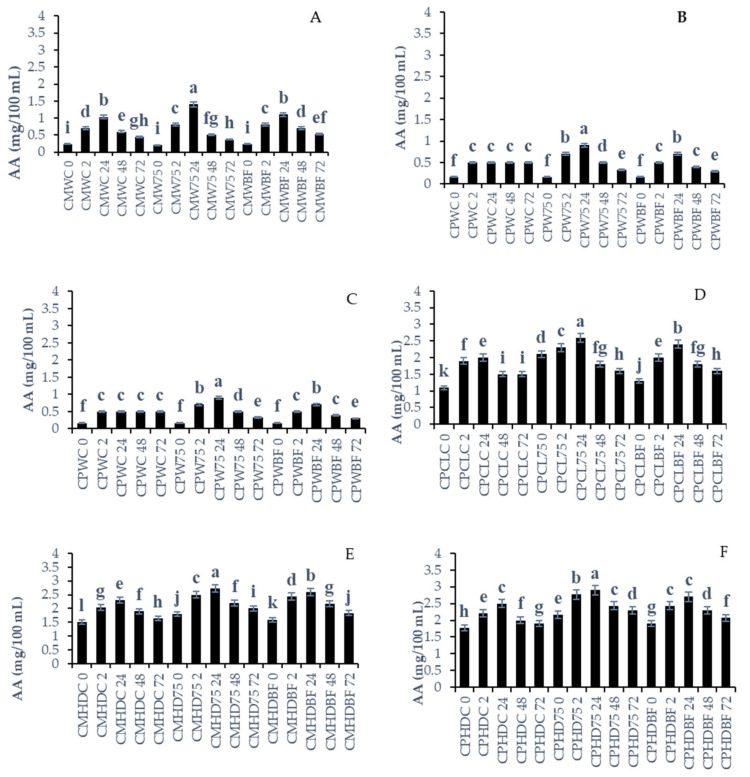
Changes in ascorbic acid concentration during fermentation with different lactobacillus strains on pumpkin leaf (*Cucurbita moschata or C. pepo)* and melon smoothies. (**A**) *Cucurbita moschata* and Watermelon smoothies fermented with LABs; (**B**) *Cucurbita pepo* + Watermelon LABS smoothies fermented with LABs; (**C**) *Cucurbita moschata* + Cantaloupe smoothies fermented with LABs; (**D**) *Cucurbita pepo* + Cantaloupe smoothies fermented with LABs; (**E**) *Cucurbita moschata* + Honeydew smoothies fermented with LABs; (**F**) *Cucurbita pepo* + Honeydew smoothies fermented with LABs. Mean values were calculated based on three replicate samples. Different letters in the same bar refer to statistical difference at *p*  <  0.05 according to Fisher’s protected LSD test. *Keys: Cucurbita moschata* + Watermelon (CMWC), *Cucurbita moschata* + Watermelon + *L75* (CMW75), *Cucurbita moschata* + Watermelon + *B. longum* (CMWBF), *Cucurbita pepo* + Watermelon Control (CPWC), *Cucurbita pepo* + Watermelon + *L75* (CPW75), *Cucurbita pepo* + Watermelon + *B. longum* (CPWBF), *Cucurbita moschata* + Cantaloupe Control (CMCLC), *Cucurbita moschata* + Cantaloupe + *L75* (CMCL75), *Cucurbita moschata* + Cantaloupe + *L75* (CMCL75), *Cucurbita pepo* + Cantaloupe Control (CPCLC), *Cucurbita pepo* + Cantaloupe + *L75* (CPCL75), *Cucurbita pepo* + Cantaloupe + *B. longum* (CPCLBF), *Cucurbita moschata* + Honeydew Control (CMHDC), *Cucurbita moschata* + Honeydew + *L75* (CMHD75), Cucurbita moschata + Honeydew + *B. longum* (CMHDBF), *Cucurbita pepo* + Honeydew Control (CPHDC), *Cucurbita pepo* + Honeydew + *L75* (CMHD75), and *Cucurbita pepo* + Honeydew + *B. longum* (CPHDBF).

Its electron-donating potential makes l-ascorbic acid (vitamin C) an effective antioxidant due to its nutritional and clinical properties [41]. There was a significant increase in ascorbic acid content in the following mixtures: *C. moschata* + Watermelon + *L75* (CMW75), *C. pepo* + Watermelon + *L75* (CPW75), *C. moschata* + Cantaloupe + *L75* (CMCL75), *C. pepo* + Cantaloupe + *L75* (CPCL75), *C. moschata* + Honeydew + *L75* (CMHD75), and *C. pepo* + Honeydew + *L75* (CPHD75) (Figure 2). These mixtures showed the highest concentration of ascorbic acid after 24 h of fermentation compared to the control groups and those inoculated and fermented with *B. longum* at 24, 48, and 72 h. The ascorbic acid content of all melon types and *C. moschata* or *C. pepo* fermented with *L75* or *B. longum* was significantly (*p* < 0.05) reduced at 72 h. Inoculated smoothies containing *B. longum* showed higher concentrations of AA after 24 h compared to their respective control groups. Specifically, the smoothies *C. pepo* + Watermelon + *B. longum* (CPWBF), *C. pepo* + Cantaloupe + *B. longum* (CPCLBF), and *C. moschata* + Honeydew + *B. longum* (CMHDBF) exhibited higher AA concentrations. However, the decrease in vitamin C content during the fermentation of the three types of melon and *C. pepo* and *C. moschata* leaf smoothies by *L75* and *B. longum* (*p* > 0.05) was consistent. This supports the hypothesis that *L75* and *B. longum* can consume ascorbic acid independently. Based on our data, we disagree with Hashemi et al. [42], who found that the concentration of ascorbic acid in *Citrus limetta* juice was not altered after fermentation with *L. plantarum LS5*. The ascorbic acid concentrations can be affected by storage and pasteurization treatment temperatures [42]. In their [39] investigation, the pasteurization temperature was at 80 °C for 5 min and stored frozen (−20 °C for 2 weeks) before analysis, and this could have affected the concentration of AA. On the other hand, the LABs could have metabolized the AA from the food matrix [42]. 

Furthermore, after 24 h of fermentation, *C. moschata* + Watermelon + *B. longum* (CMWBF) and *C. moschata* + Watermelon Control (CMWC) showed similar levels of AA. Similarly, after 24 h of fermentation, *C. moschata* + Cantaloupe + *B. longum* (CMCLBF) and *C. moschata* + Cantaloupe Control (CMCLC) exhibited similar levels of AA. Likewise, *C. moschata* + Honeydew + *B. longum* (CMHDBF) and *C. moschata* + Honeydew Control (CMHDC), after 24 h of fermentation, showed similar levels of AA. Even though ascorbic acid is abundant in melon and pumpkin leaf smoothies, the fermentation can slow down the loss of ascorbic acid, and a similar observation was shown by Xu et al. [42]. Ascorbic acid content rose in citrus ‘Tarocco’ juice, rising to 295 ± 25.3 g L^−1^ when fermented by *L. brevis* [43]. Furthermore, according to Xu et al. [43], citrus juice fermentation accumulated a high concentration of ascorbic acid (above 300 mg/L) with *L. brevis* 182 and *L. paracasei* 502.

Fermentation decreases vitamin C content due to its instability and easy oxidative decomposition, as well as the metabolic utilization of lactic acid bacteria [44]. In smoothies and juices, ascorbic acid degradation occurs due to the presence of ascorbic acid oxidase, which is not completely destroyed by pasteurization [44]. Several factors influence ascorbic acid stability, including redox state, metal ion concentration, and pH [44]. Furthermore, Urbienė and Mitkute [45] found that the growth of LABs and the increase in their population throughout fermentation had a minimal impact on changes in ascorbic acid, since oxidation was the main cause of degradation.

### 3.6. Sensory Properties

**Figure 3:**
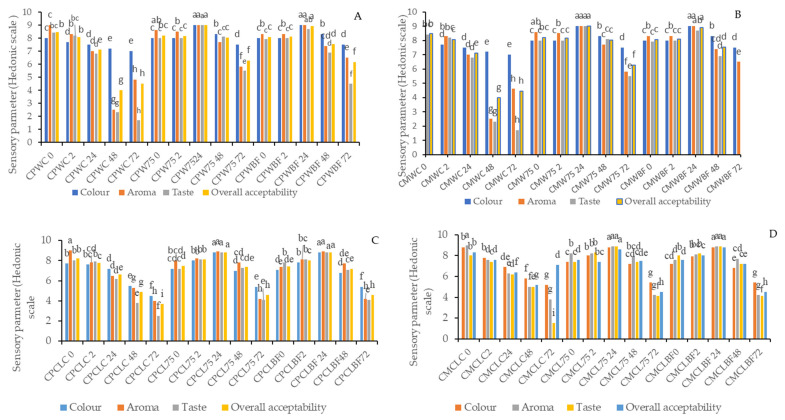

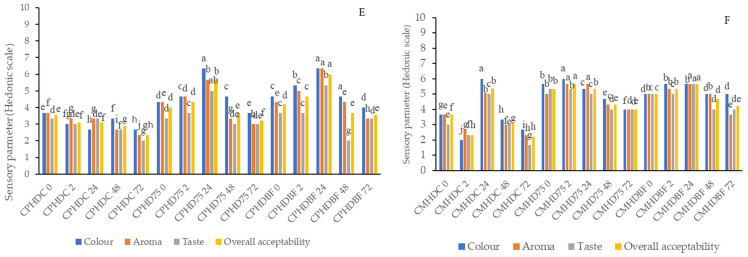
Sensory properties of pumpkin leaf (*Cucurbita moschata* or *C. pepo*) and melon smoothies fermented with different lactobacillus strains. Different letters in the same bar refer to statistical difference at *p*  <  0.05 according to Fisher’s protected LSD test. (**A**,**C**) *Cucurbita moschata* + Cantaloupe smoothies fermented with LABs; (**B**,**E**) *Cucurbita pepo* + Cantaloupe smoothies fermented with LABs; (**D**,**F**) *Cucurbita pepo* + Watermelon smoothies fermented with LABs; *Keys: Cucurbita moschata* + Watermelon (CMWC), *Cucurbita moschata* + Watermelon + *L75* (CMW75), *Cucurbita moschata* + Watermelon + *B. longum* (CMWBF), *Cucurbita pepo* + Watermelon Control (CPWC), *Cucurbita pepo* + Watermelon + *L75* (CPW75), *Cucurbita pepo* + Watermelon + *B. longum* (CPWBF), *Cucurbita moschata* + Cantaloupe Control (CMCLC), *Cucurbita moschata* + Cantaloupe + *L75* (CMCL75), *Cucurbita moschata* + Cantaloupe + *L75* (CMCL75), *Cucurbita pepo* + Cantaloupe Control (CPCLC), *Cucurbita pepo* + Cantaloupe + *L75* (CPCL75), *Cucurbita pepo* + Cantaloupe + *B. longum* (CPCLBF), *Cucurbita moschata* + Honeydew Control (CMHDC), *Cucurbita moschata* + Honeydew + *L75* (CMHD75),Cucurbita moschata + Honeydew + *B. longum* (CMHDBF), *Cucurbita pepo* + Honeydew Control (CPHDC),*Cucurbita pepo* + Honeydew + *L75* (CMHD75) and *Cucurbita pepo* + Honeydew + *B. longum* (CPHDBF).

In Figure 3, the sensory qualities of smoothies are presented. The sensory qualities of smoothies made with three types of melon and pumpkin leaves were affected by fermentation. The overall quality was determined based on the color, aroma, and taste of all the samples. It was observed that smoothies fermented with *L75* and *B. longum* retained color, aroma, taste, and acceptability 24 h after fermentation, similar to non-inoculated smoothies at 0 h. After 24 h of fermentation, CPCL75, CPCLBF, CMCL75, and CMCLBF achieved an overall quality score of 8.8. The CMW75, CMWBF, CPW75, and CPWBF fermented smoothies scored 9.0 and 8.9 for overall quality after 24 h. Moreover, CMHD75 and CMHDBF showed overall quality scores of 5.7 after 2 h of fermentation. Similarly, CPHD75 and CPHDBF showed scores of 2.6 and 5.3 after 24 h of fermentation, respectively. Some fermented smoothies from Honeydew melon scored around 5.3 and 5.7 (neither liked nor disliked) for overall acceptability, while the non-inoculated Honeydew smoothies scored around 2.3 (extremely disliked). Increased fermentation time adversely affected the sensory properties of the color, taste, and aroma, while negatively impacting the overall acceptability of these smoothies. Due to a change in the metabolic process of the cultures, increased fermentation time resulted in alterations in the smoothies’ contents that negatively affected the final product’s aroma, color, and taste. Lactic acid bacteria (LABs) primarily utilize glucose and fructose to produce organic acids and specific aromatic compounds that contribute to the flavor of the food [46]. According to the results, panelists may have been influenced by sweetness, pleasant aroma (due to volatile compounds), and color in making their decisions. In a sensory evaluation, reduced taste could be due to sourness associated with organic acids, while sweetness is linked to sugars such as glucose and fructose [46]. Sensory evaluation further confirmed the higher sugar (TSS) consumption and organic acid (lactic acid) production during 72 h fermentation.

### 3.7. Total Phenols

**Figure 4:**
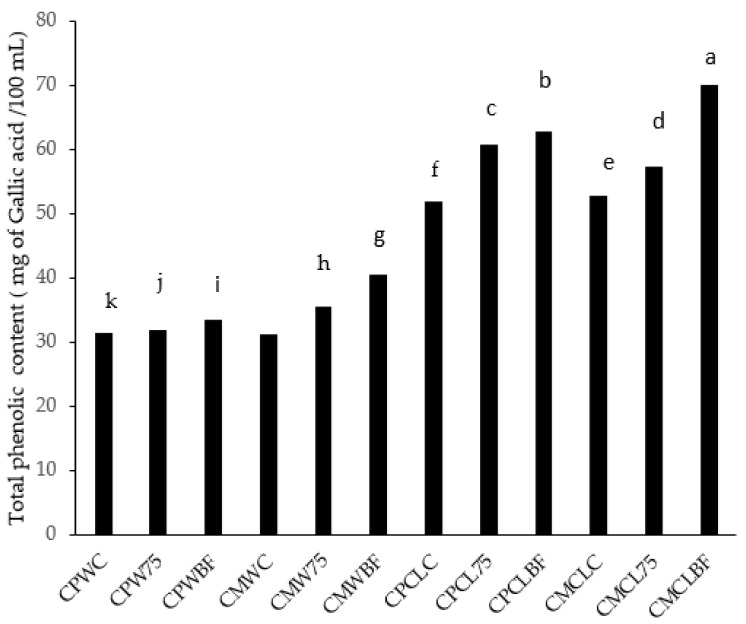
Changes in total phenolic content during fermentation with different lactobacillus strains on pumpkin leaf (*Cucurbita moschata or C. pepo)* and melon smoothies. Mean values were calculated based on three replicate samples. Different letters in the same bar refer to statistical difference at *p*  <  0.05 according to Fisher’s protected LSD test. *Cucurbita pepo* + Watermelon Control (CPWC), *Cucurbita pepo* + Watermelon + *L75* (CPW75), *Cucurbita pepo* + Watermelon + *B. longum* (CPWBF), *Cucurbita moschata* + Watermelon Control (CMWC), *Cucurbita moschata* + Watermelon + *L75* (CMW75), *Cucurbita moschata* + Watermelon + *B. longum* (CMWBF), *Cucurbita moschata* + Cantaloupe Control (CMCLC), *Cucurbita moschata* + Cantaloupe + *L75* (CMCL75), *Cucurbita moschata* + Cantaloupe + *B. longum* (CMCLBF), *Cucurbita Moschata* + Cantaloupe Control (CMCLC), *Cucurbita Moschata* + Cantaloupe+ *L75* (CMCL75), and *Cucurbita Moschata* + Cantaloupe + *B. longum* (CMCLBF).

Figure 4 shows the effect of fermentation on TPC, and it increased between 31.45 mg GAE/100 mL and 70.17 mg GAE/100 mL when Cantaloupe and Watermelon were fermented with *L75* or *B. Longum*. When fermented with *B. Longum*, both *C. pepo* + Cantaloupe + *B. longum* (CPCLBF) and *C. moschata* + Cantaloupe + *B. longum* (CMCLBF) showed the highest level of phenolics (62.91 mg GAE/100 mL; 70.17 mg GAE/100 mL) compared to *C. moschata* + Watermelon and *C. pepo* + Watermelon fermented with *B. Longum* and *L75* (CMW75, CMWBF, CPW75, and CPWBF), as well as *C. moschata* + Cantaloupe (CMCL75) and *C. pepo* + Cantaloupe fermented with *L75* (CPCL75). The TPC was increased 1.33-fold by fermenting *C. moschata* + Cantaloupe with *B. longum* compared to their control samples. In general, TPC increased from 1.01- to 1.33-fold when fermented with LAB strains compared with their control samples. It is thought that the increase in TPC during fermentation is due to the ability of *L75* and *B longum* to deglycosylate glycosylated phenolic compounds in plant cells [47]. As substrates in food fermentation, polyphenols are hydrolyzed into smaller phenolic compounds with higher bioactivity and bioavailability by polyphenol-associated enzymes (tannases, esterases, phenolic acid decarboxylases, and glycosidases) [48]. Lactic acid bacteria secrete tannases, esterases, phenolic acid decarboxylases, or glycosidases during lactic acid fermentation and release the bound phenols to free phenols. In fermented foods, this biotransformation increases the amount and type of free phenols [48]. Different polyphenols undergo different biotransformation pathways by polyphenol-associated enzymes secreted by different microorganisms [48]. Furthermore, *Bifidobacterium longum* can deglycosylate a wide range of substrates due to their genome-encoded β-glycosidases [47].

### 3.8. Total Carotenoids (TCCs), Lutein, and β Carotene

In *C. pepo* + Cantaloupe fermented with *L75* (CPCL75), TCCs showed the highest concentration (26.14 mg/100 mL), followed by *C. pepo* + Cantaloupe fermented with *B. longum* (CPCLBF) (23.18 mg/100 mL), compared to all other fermented and unfermented smoothies (Table 2). Compared with the control counterparts, *C. pepo* and Cantaloupe fermented with *L75* (CPCL75) or *B. longum* (CPCLBF) increased their TCC content 1.85- and 1.64-fold, respectively. Contrary to this, fermentation with *L75* increased the TCCs for *C. moschata* and Watermelon (CMW75) 1.22-fold, but only 1.16-fold with *B. longum* (CMWBF). Moreover, when *C. pepo* and Cantaloupe are combined, fermentation with *L75* and *B. longum* results in a 1.85-fold and 1.64-fold increase, respectively, in TCCs as compared to their control counterparts. Antognoni et al. [49] showed an increase in the carotenoid content of wheat dough after fermentation with *L. plantarum* (29DAN, 83DAN). Among all fermented smoothies, *C. pepo* + Cantaloupe smoothie fermented with *L75* (CPCL75) showed the highest concentration of lutein (2.53 mg/100 mL), trans-β-carotene (620.37 mg/100 mL), and cis-β-carotene (28.20 mg/100 mL). However, *C. moschata* + Watermelon fermented with *B. longum* (CMWBF) demonstrated a 1.46-fold increase in lutein compared to their unfermented control samples. Watermelon and *C. pepo* fermented with *L75* (CPW75) or *B. longum* (CPWBF) showed a 3.26- and 3.09-fold increase, respectively, in trans-β-carotene compared to their control samples. Similar trends in the increase in cis β-carotene were observed as well. These results show that the addition of *lactobacillus* or *bifidobacterium* to Watermelon and *C. pepo* can increase the concentration of beneficial carotenoids. Meanwhile, *C. moschata* combined with Watermelon and fermented with *L75* (CMW75) showed a 7.57-fold increase in cis-β-carotene and a 4.97-fold increase in trans-β-carotene compared to the control samples. Also, *C. pepo* + Cantaloupe fermented with *L75* (CPCL75) showed a 1.57- and 2.41-fold increase in trans and cis-β-carotenes. Contrary to our findings, Szutowska et al. [50] showed that curly kale juice fermented with LAB strains reduced the amount of lutein and beta-carotene, respectively, by 40% and 34.8%, while the amount of carotenoid decreased by 17–31%. In addition, Odongo et al. [51] reported that Ethiopian kale fermented with LAB strains lost 98% of its lutein, which was also 75% of its carotenoids. Demir et al. [52] reported an increase in carrot juice carotene content following fermentation by *L. plantarum,* similar to our findings. Carotenoid content decreases during lactic acid fermentation due to the formation of volatile derivatives, which contribute to the flavor and aroma of fermented foods [53]. Meanwhile, Oloo et al. [54] investigated the influence of lactic fermentation (*L. plantarum* MTCC 1407 at 25 ± 2 °C for 48 h) on the accumulation of β-carotene in three varieties of orange-fleshed sweet potatoes (Zapallo, Nyathiodiewo, and SPK004/06) from Kenya and demonstrated the elevation of carotene content by 93.97%.

Bartkiene et al. [55] showed tomato varieties Ronaldo and Cunero fermented with *L sakei* increased total carotenoids levels by 41.1 and 33.6%, respectively, compared to the untreated samples. However, the observed significant increase in this carotene could be due to a variety of reasons. In the fermentation of melon and pumpkin leaves, enzymatic activities and microbiological conversions (macromolecular changes) are involved, and several strains of *L. plantarum* produce carotenoids to protect against oxidative stress, or fermentation-induced structural changes could have enhanced the extraction of carotenoids [56]. In our research and that of other researchers, we found that factors such as carotenoid type, matrix, and fermentation conditions can greatly affect the bioavailability of carotenoid components. However, a comprehensive metabolomics study of carotenoids and their derivatives before and after fermentation with lactic acid has not been conducted. Carotenoid compounds contain health benefits due to their antioxidant properties, which are highly effective radical quenchers [56]. The prevalence of all-trans β-carotene in tissues and serum indicates that it may be the preferred form of β-carotene [57]. The smoothies made from various types of melon and pumpkin leaves were found to contain between 10 and 60 mg of lutein per 10 mL, while 100 mL of these smoothies contained about 2500 µg RAE of vitamin A. This suggests that consuming a cup of these smoothies will help meet the general daily recommendation of 6–10 mg of lutein and 900–700 µg RAE of vitamin A for adult men and women, respectively.

*Cucurbita pepo* + Watermelon Control (CPWC), *Cucurbita pepo* + Watermelon + *L75* (CPW75), *Cucurbita pepo* + Watermelon + *B. longum* (CPWBF), *Cucurbita moschata* + Watermelon Control (CMWC), *Cucurbita moschata* + Watermelon + *L75* (CMW75), *Cucurbita moschata* + Watermelon + *B. longum* (CMWBF), *Cucurbita moschata* + Cantaloupe Control (CMCLC), *Cucurbita moschata* + Cantaloupe + *L75* (CMCL75), *Cucurbita moschata* + Cantaloupe + *B. longum* (CMCLBF), *Cucurbita Moschata* + Cantaloupe Control (CMCLC), *Cucurbita Moschata* + Cantaloupe+ *L75* (CMCL75), and *Cucurbita Moschata* + Cantaloupe + *B. longum* (CMCLBF).

### 3.9. Antioxidant Activities

*Cucurbita moschata* + Watermelon + *L75* (CMW75) showed the highest antioxidant power (FRAP) (9.89 mM TEAC/mL) followed by *Cucurbita pepo* + Cantaloupe + *L75* (CPCL75) (7.76 mM TEAC/mL) and *Cucurbita moschata* + Cantaloupe + *L75* (7.40 mM TEAC/mL). CMW75 also showed a 1.23-fold increase in TPC compared to their unfermented control samples (Table 3). The increased FRAP activity could be due to the ability of antioxidant properties and phenols to typically scavenge free radicals [58], chelate metal ions, and act as reduction agents [58] in the fermented smoothies. Moreover, fermentation can produce new phenolic compounds while improving the concentration of the present compounds and enhancing the contribution of free soluble antioxidants.

*Cucurbita pepo* + Cantaloupe + *L75* (CPCL75) showed the highest DPPH radical scavenging activity (1.18 IC50 µL/mL) followed by *Cucurbita moschata* + Cantaloupe + *L75* (CMCL75) (2.66 IC50 µL/mL) (Table 3). In general, *L75*-fermented CMW75, CPCL75, and CMCL75 smoothies showed higher DPPH radical scavenging activity than *B. longum* fermented smoothies. There is, however, a similar level of DPPH scavenging activity in smoothies made with CMW75 and CMWBF. In addition, the melon and pumpkin leaf smoothies fermented with *L75* showed the highest ascorbic acid content. DPPH strongly correlates with AA; the increased DPPH scavenging activity could be attributed to the high concentration of AA available in the smoothies as it scavenges the free radicals, thus improving DPPH potency [12]. Additionally, a reduction in pH, as observed in Section 3.3, enhances the durability of ascorbic acid and decelerates its conversion to dehydroascorbic acid, thereby aiding in an increased DPPH scavenging ability [59].

A higher level of ABTS scavenging activity was observed in CMCL75 smoothies followed by CMCLBF smoothies (Table 3). Overall, the melon and pumpkin leaf smoothie combinations fermented with *L75* had significantly (*p* < 0.05) greater antioxidant power (FRAP) and DPPH scavenging activity than smoothies fermented with *B. longum*. Above all, the increased levels of antioxidant properties in these smoothies may be attributed to the higher levels of phytochemicals such as phenolics and AA for FRAP and DPPH and carotenoids for ABTS that are produced through microbial hydrolysis or biotransformation, leading to the breakdown of antioxidant polysaccharides and peptides in the smoothies [60]. Different chemical structures of the radicals and the mechanism of action of the molecules, as well as the type of phenolic compounds present in the smoothies, contribute to the variation in antioxidant scavenging potential. Carotenoids’ structures influence their antioxidative activity. The carbonyl and hydroxyl groups play a significant role in the carotenoids’ quenching capacity, which is due to their affinity to reactive oxygen species (ROS). Thus, carotenoids can be enhanced by adding more hydroxyl groups or by adding conjugated double bonds (C=C and C=O) to increase their antioxidant activity [61]. Carotenoids donate electrons to ROS and create carotenoid–radical cations by oxygenation, thus lowering the oxidation state of the ROS [62]. Also, carotenoids scavenge reactive oxygen species through the hydrogen atom transfer mechanism in which a proton and electron are moved between two substrates during a chemical reaction [48]. 

*Cucurbita pepo* + Watermelon Control (CPWC), *Cucurbita pepo* + Watermelon + *L75* (CPW75), *Cucurbita pepo* + Watermelon + *B. longum* (CPWBF), *Cucurbita moschata* + Watermelon Control (CMWC), *Cucurbita moschata* + Watermelon + *L75* (CMW75), *Cucurbita moschata* + Watermelon + *B. longum* (CMWBF), *Cucurbita moschata* + Cantaloupe Control (CMCLC), *Cucurbita moschata* + Cantaloupe + *L75* (CMCL75), *Cucurbita moschata* + Cantaloupe + *B. longum* (CMCLBF), *Cucurbita Moschata* + Cantaloupe Control (CMCLC), *Cucurbita Moschata* + Cantaloupe+ *L75* (CMCL75), and *Cucurbita Moschata* + Cantaloupe + *B. longum* (CMCLBF). 

## 4. Conclusions

Smoothies made from fruits and vegetables are rich in vitamins, minerals, and bioactive compounds, such as carotenoids, along with fiber. Fermented products can also reduce post-harvest losses and food waste while improving food security. Probiotic non-dairy products are commonly produced by fermenting fruit and vegetable commodities by lactic acid bacteria to enhance their health-related functional characteristics and bioactive compounds. Developing non-dairy probiotic smoothies enriched with bioactive compounds opens new perspectives, as functional compounds contained in fermented fruit and indigenous vegetable products emphasize the phytonutrient content. This study comprehensively examined the impact of two different lactic acid bacterium (LAB) strains inoculation on the changes in physicochemical parameters, total phenols, total carotenoids and the components of carotenoids, and antioxidant activity in different types of melon and pumpkin leaf smoothies. This study confirmed the potential of fermenting Cantaloupe and pumpkin leaves together with the *L75* strain to produce non-dairy functional products.

Furthermore, these smoothies provide a significant amount of ascorbic acid, lutein, and β-carotene. Adding them to our daily diets will contribute to diversifying our diet, while also supporting overall health and meeting daily nutrient requirements. Additionally, fermentation with probiotics that produce high-antioxidant carotenoids can deliver substantial health benefits by enhancing digestion and reducing oxidative stress in the body, preventing cellular damage. However, further research is needed to enhance the application of fermentation to produce microbial carotenoids by obtaining a deeper understanding of less common carotenoid types that could be beneficial to consumers.

## Figures and Tables

**Table 1:** Changes in physicochemical parameters during fermentation with different lactobacillus strains on pumpkin leaf (*Cucurbita moschata or C. pepo)* and melon smoothies.

*Cucurbita moschata* Leaf and Watermelon Smoothies Fermented with LABs				
	Fermentation Time (h)	TSS (°Brix)	pH	TA (Equivalent Lactic Acid g/100 g)
CMWC	0	9.63 ± 0.058 ^a,^*	5.69 ± 0.007 ^a^	1.333 ± 0.058 ^k^
CMWC	2	9.20 ± 0.100 ^b^ (−4.5%) **	5.54 ± 0.010 ^d^	1.66 ± 0.006 ^j^ (+24.5%) ***
CMWC	24	8.50 ± 0.100 ^d^ (−11.7%)	5.47 ± 0.000 ^e^	1.67 ± 0.116 ^j^ (+25.3%)
CMWC	48	8.10 ± 0.173 ^f^ (−15.9%)	4.94 ± 0.006 ^h^	1.77 ± 0.058 ^i^ (+32.8%)
CMWC	72	7.86 ± 0.058 ^g^ (−18.4%)	4.84 ± 0.006 ^i^	2.30 ± 0.000 ^g^ (+72.5%)
CMW75	0	9.20 ± 0.000 ^b^	5.61 ± 0.000 ^c^	1.83 ± 0.058 ^h^
CMW75	2	8.36 ± 0.058 ^de^ (−9.1%)	5.28 ± 0.000 ^g^	3.33 ± 0.058 ^f^ (+81.9%)
CMW75	24	7.90 ± 0.100 ^g^ (−14.1%)	4.34 ± 0.000 ^h^	4.26 ± 0.153 ^e^ (+132.8%)
CMW75	48	7.56 ± 0.116 ^h^ (−17.8%)	3.89 ± 0.000 ^k^	5.53 ± 0.058 ^c^ (+202.2%)
CMW75	72	7.33 ± 0.058 ^i^ (−20.3%)	3.76 ± 0.006 ^m^	5.80 ± 0.000 ^b^ (+216.9%)
CMWBF	0	9.00 ± 0.100 ^c^	5.63 ± 0.06 ^b^	1.86 ± 0.058 ^h^
CMWBF	2	8.33 ± 0.058 ^e^ (−7.4%)	5.31 ± 0.06 ^f^	3.36 ± 0.018 ^f^ (+80.6%)
CMWBF	24	7.567 ± 0.058 ^h^ (−15.9%)	4.30 ± 0.010 ^j^	4.66 ± 0.056 ^d^ (+150.5%)
CMWBF	48	7.16 ± 0.153 ^j^ (−20.4%)	3.88 ± 0.056 ^k^	5.76 ± 0.078 ^b^ (+209.7%)
CMWBF	72	6.80 ± 0.010 ^k^ (−24.4%)	3.76 ± 0.016 ^m^	5.92 ± 0.070 ^a^ (+218.3%)
*Cucurbita pepo* leaf and Watermelon smoothies fermented with LABs				
	Fermentation Time (h)	TSS (°Brix)	pH	TA (Equivalent Lactic Acid g/100 g)
CPWC	0	8.03 ± 0.058 ^a^	5.34 ± 0.05 ^a^	1.06 ± 0.116 ^m^
CPWC	2	7.66 ± 0.018 ^b^ (−4.6%)	4.98 ± 0.006 ^c^	1.53 ± 0.058 ^l^ (+44.3%)
CPWC	24	7.30 ± 0.100 ^d^ (−9.1%)	4.81 ± 0.012 ^d^	1.70 ± 0.100 ^k^ (+60.4%)
CPWC	48	6.90 ± 0.0200 ^f^ (−14.1%)	4.75 ± 0.006 ^e^	1.98 ± 0.058 ^j (^+86.8%)
CPWC	72	6.50 ± 0.040 ^h^ (−19.1%)	4.70 ± 0.022 ^f^	2.30 ± 0.010 ^i^ (+117.0%)
(CPW75)	0	7.53 ± 0.058 ^c^	5.29 ± 0.050 ^b^	1.80 ± 0.010 ^k^
CPW75	2	7.36 ± 0.068 ^d^ (−2.3%)	4.66 ± 0.012 ^g^	2.86 ± 0.058 ^f^ (+58.9%)
CPW75	24	7.03 ± 0.059 ^e^ (−6.6%)	4.08 ± 0.010 ^h^	4.83 ± 0.058 ^d^ (+168.3%)
CPW75	48	6.43 ± 0.048 ^h^ (−14.6%)	3.71 ± 0.010 ^i^	5.46 ± 0.058 ^c^ (+203.3%)
CPW75	72	6.00 ± 0.010 ^j^ (−20.3%)	3.62 ± 0.006 ^j^	6.48 ± 0.116 ^a^ (+260.0%)
CPWBF	0	7.63 ± 0.018 ^b^	5.30 ± 0.074 ^b^	1.53 ± 0.028 ^l^
CPWBF	2	7.36 ± 0.055 ^d^ (−3.5%)	4.71 ± 0.066 ^e^	2.26 ± 0.018 ^h^ (+47.7%)
CPWBF	24	6.90 ± 0.060 ^f^ (−9.6%)	4.08 ± 0.0076 ^h^	4.53 ± 0.098 ^e^ (+196.1%)
CPWBF	48	6.60 ± 0.080 ^g^ (−13.5%)	3.71 ± 0.036 ^i^	5.40 ± 0.0240 ^c^ (+252.9%)
CPWBF	72	6.20 ± 0.008 ^i^ (−18.7%)	3.65 ± 0.010 ^j^	5.98 ± 0.012 ^b^ (+290.8%)
*Cucurbita moschata* leaf and Cantaloupe smoothies fermented with LABs				
	Fermentation Time (h)	TSS (%)	pH	TA (Equivalent Lactic acid g/100 g
CMCLC	0	6.30 ± 0.01 ^a^	6.66 ± 0.010 ^a^	1.20 ± 0.050 ^m^
CMCLC	2	5.70 ± 0.02 ^b^ (−9.5%)	6.44 ± 0.015 ^c^	1.35 ± 0.150 ^l^ (+12.5%)
CMCLC	24	5.13 ± 0.06 ^d^ (−18.6%)	6.38 ± 0.000 ^d^	1.67 ± 0.120 ^k^ (+39.2%)
CMCLC	48	4.73 ± 0.06 ^ef^ (−24.9%)	6.09 ± 0.006 ^e^	1.69.100 ^k^ (+40.8%)
CMCLC	72	4.20 ± 0.10 ^g^ (−33.3%)	5.85 ± 0.000 ^f^	1.82 ± 0.01 ^j^ (+51.7%)
CMCL75	0	5.73 ± 0.12 ^b^	6.52 ± 0.002 ^b^	2.33 ± 0.050 ^h^
CMCL75	2	4.77 ± 0.12 ^e^ (−16.7%)	5.27 ± 0.006 ^h^	3.87 ± 0.050 ^f^ (+66.1%)
CMCL75	24	4.23 ± 0.06 ^g^ (−26.2%)	4.32 ± 0.000 ^i^	4.21 ± 0.100 ^d^ (+80.7%)
CMCL75	48	3.80 ± 0.10 ^h^ (−33.7%)	4.04 ± 0.000 ^k^	4.50 ± 0.010 ^b^ (+93.1%)
CMCL75	72	3.53 ± 0.06 ^I^ (−38.4%)	3.77 ± 0.001 ^n^	4.97 ± 0.120 ^a^ (+113.3%)
CMCLBF	0	5.53 ± 0.06 ^c^	6.56 ± 0.006 ^b^	2.23 ± 0.116 ^i^
CMCLB	2	4.63 ± 0.06 ^f^ (−19.4%)	5.32 ± 0.006 ^g^	3.50 ± 0.030 ^g^ (+56.9%)
CMCLBF	24	4.27 ± 0.05 ^g^ (−22.8%)	4.27 ± 0.000 ^j^	4.14 ± 0.100 ^e^ (+85.6%)
CMCLBF	48	3.83 ± 0.06 ^h^ (−30.7%)	4.19 ± 0.000 ^l^	4.30 ± 0.013 ^c^ (+92.8%)
CMCLBF	72	3.60 ± 0.01 ^I^ (−34.9%)	3.81 ± 0.006 ^m^	4.57 ± 0.058 ^b^ (+104.9%)
*Cucurbita pepo* leaf and Cantaloupe melon smoothies fermented with LABs				
	Fermentation Time (h)	TSS (%)	pH	TA (Equivalent Lactic Acid g/100 g
CPCLC				
CPCLC	0	5.3 ± 0.01 ^a^	6.43 ± 0.001 ^a^	1.53 ± 0.060 ^l^
CPCLC	2	5.1 ± 0.02 ^b^ (−3.8%)	6.24 ± 0.002 ^d^	1.59 ± 0.060 ^l^ (+3.9%)
CPCLC	24	4.9 ± 0.01 ^e^ (−7.5%)	5.87 ± 0.001 ^e^	1.67 ± 0.050 ^k^ (+9.1%)
CPCLC	48	4.9 ± 0.03 ^e^ (−7.5%)	5.76 ± 0.004 ^f^	1.74 ± 0.060 ^j^ (+13.7%)
CPCLC	72	4.4 ± 0.10 ^I^ (−17.0%)	5.49 ± 0.003 ^g^	1.78 ± 0.040 ^j^ (+16.3%)
CPCL75	0	5.0 ± 0.06 ^d^	6.42 ± 0.001 ^c^	2.03 ± 0.030 ^h^
CPCL75	2	4.8 ± 0.03 ^f^ (−4.0%)	5.43 ± 0.009 ^h^	3.97 ± 0.120 ^d^ (+95.6%)
CPCL75	24	4.6 ± 0.04 ^g^ (−8.0%)	4.32 ± 0.002 ^i^	4.10 ± 0.020 ^c^ (+102.0%)
CPCL75	48	4.500 ± 0.02 ^h^ (−10.0%)	4.24 ± 0.003 ^i^	4.27 ± 0.060 ^b^ (+110.0%)
CPCL75	72	4.000 ± 0.03 ^j^ (−20.0%)	3.78 ± 0.006 ^k^	4.43 ± 0.060 ^a^ (+118.2%)
CPCLBF	0	5.03 ± 0.06 ^c^	6.43 ± 0.002 ^b^	1.90 ± 0.030 ^i^
CPCLBF	2	4.8 ± 0.01 ^f^ (−4.6%)	5.40 ± 0.001 ^h^	2.53 ± 0.070 ^g^ (+33.2%)
CPCLBF	24	4.5 ± 0.01 ^h^ (−10.5%)	4.27 ± 0.001 ^i^	3.00 ± 0.050 ^f^ (+57.9%)
CPCLBF	48	4.5 ± 0.02 ^h^ (−10.5%)	4.10 ± 0.002 ^j^	3.78 ± 0.060 ^e^ (+98.9%)
CPCLBF	72	3.97 ± 0.03 ^j^ (−21.1%)	3.81 ± 0.001 ^k^	3.90 ± 0.070 ^d^ (+105.2%)
*Cucurbita moschata* leaf and Honeydew melon smoothies fermented with LABs				
	Fermentation time (h)	TSS (%)	pH	TA (Equivalent Lactic Acid g/100 g
CMHDC	0	5.80 ± 0.01 ^a^	6.44 ± 0.006 ^a^	1.06 ± 0.05 ^k^
CMHDC	2	4.90 ± 0.02 ^c^ (−15.5%)	6.37 ± 0.005 ^c^	1.70 ± 0.08 ^j^ (+60.4%)
CMHDC	24	4.30 ± 0.01 ^f^ (−25.9%)	5.93 ± 0.001 ^d^	1.78 ± 0.01 ^j^ (+67.9%)
CMHDC	48	4.00 ± 0.03 ^h^ (−31.0%)	5.80 ± 0.004 ^e^	2.01 ± 0.06 ^I^ (+89.6%)
CMHDC	72	3.87 ± 0.05 ^I^ (−33.3%)	5.82 ± 0.006 ^e^	2.06 ± 0.06 ^I^ (94.3%)
CMHD75	0	5.40 ± 0.01 ^b^	6.40 ± 0.002 ^b^	2.46 ± 0.05 ^h^
CMHD75	2	4.50 ± 0.01 ^e^ (−16.7%)	5.56 ± 0.001 ^f^	3.30 ± 0.06 ^g^ (+34.1%)
CMHD75	24	3.86 ± 0.05 ^i^ (−28.5%)	4.20 ± 0.002 ^h^	3.90 ± 0.02 ^f^ (+58.5%)
CMHD75	48	3.40 ± 0.01 ^l^ (−37.0%)	3.94 ± 0.006 ^i^	4.36 ± 0.06 ^e^ (+77.2%)
CMHD75	72	3.10 ± 0.02 ^n^ (−52.6%)	3.78 ± 0.001 ^k^	5.70 ± 0.10 ^a^ (+131.7%)
CMHDBF	0	4.80 ± 0.01 ^d^	6.36 ± 0.002 ^c^	2.46 ± 0.08 ^h^
CMHDBF	2	4.20 ± 0.02 ^g^ (−12.5%)	5.23 ± 0.005 ^g^	3.95 ± 0.06 ^f^ (+60.6%)
CMHDBF	24	3.80 ± 0.05 ^j^ (−20.8%)	4.19 ± 0.006 ^h^	4.90 ± 0.02 ^d^ (+99.2%)
CMHDBF	48	3.53 ± 0.05 ^k^ (−26.4%)	3.85 ± 0.001 ^j^	5.26 ± 0.05 ^c^ (+113.8%)
CMHDBF	72	3.20 ± 0.02 ^m^ (−33.3%)	3.79 ± 0.061 ^k^	5.33 ± 0.06 ^b^ (+116.7%)
*Cucurbita pepo* leaf and Honeydew melon smoothies fermented with LABs				
	Fermentation time (h)	TSS(%)	pH	TA (Equivalent Lactic Acid g/100 g
CPHDC	0	4.53 ± 0.06 ^c^	6.27 ± 0.001 ^a^	1.08 ± 0.058 ^n^
CPHDC	2	4.40 ± 0.01 ^d^ (−2.9%)	6.18 ± 0.002 ^d^	1.52 ± 0.01 ^m^ (+40.7%)
CPHDC	24	4.16 ± 0.05 ^f^ (−8.2%)	5.87 ± 0.001 ^f^	1.65 ± 0.01 ^l^ (+52.8%)
CPHDC	48	3.86 ± 0.06 ^h^ (−14.8%)	5.57 ± 0.004 ^h^	2.00 ± 0.02 ^k^ (+85.2%)
CPHDC	72	3.60 ± 0.07 ^j^ (−20.5%)	4.91 ± 0.006 ^i^	2.26 ± 0.05 ^j^ (109.3%)
CMHD75	0	5.13 ± 0.05 ^a^	6.23 ± 0.002 ^b^	3.46 ± 0.05 ^i^
CMHD75	2	4.80 ± 0.01 ^b^ (−6.4%)	5.96 ± 0.005 ^e^	5.30 ± 0.06 ^f^ (+53.2%)
CMHD75	24	4.50 ± 0.02 ^c^ (−12.3%)	4.16 ± 0.005 ^j^	6.80 ± 0.08 ^e^ (+96.5%)
CMHD75	48	4.20 ± 0.05 ^f^ (−18.1%)	4.05 ± 0.009 ^k^	7.16 ± 0.08 ^c^ (+106.9%)
CMHD75	72	4.03 ± 0.11 ^g^ (−21.4%)	4.02 ± 0.008 ^l^	7.36 ± 0.06 ^a^ (+112.7%)
CPHDBF	0	4.30 ± 0.07 ^e^	6.21 ± 0.002 ^c^	3.44 ± 0.05 ^i^
CPHDBF	2	4.20 ± 0.05 ^f^ (−2.3%)	5.76 ± 0.001 ^g^	4.70 ± 0.09 ^h^ (+36.6%)
CPHDBF	24	3.90 ± 0.06 ^h^ (−9.3%)	4.17 ± 0.006 ^j^	5.20 ± 0.03 ^fg^ (+51.1%)
CPHDBF	48	3.70 ± 0.07 ^i^ (−13.9%)	3.97 ± 0.006 ^m^	7.03 ± 0.07 ^d^ (+104.3%)
CPHDBF	72	3.10 ± 0.08 ^k^ (−27.9%)	3.81 ± 0.006 ^n^	7.20 ± 0.01 ^b^ (+109.3%)

Mean values were calculated based on three replicate samples. Different letters in the same column refer to statistical difference at *p*  <  0.05 according to Fisher’s protected LSD test. * Standard deviation; −% decrease **; +% increase ***. *Keys: Cucurbita moschata* + Watermelon (CMWC), *Cucurbita moschata* + Watermelon + *L75* (CMW75), *Cucurbita moschata* + Watermelon + *B. longum* (CMWBF), *Cucurbita pepo* + Watermelon Control (CPWC), *Cucurbita pepo* + Watermelon + *L75* (CPW75), *Cucurbita pepo* + Watermelon + *B. longum* (CPWBF), *Cucurbita moschata* + Cantaloupe Control (CMCLC), *Cucurbita moschata* + Cantaloupe + *L75* (CMCL75), *Cucurbita moschata* + Cantaloupe + *L75* (CMCL75), *Cucurbita pepo* + Cantaloupe Control (CPCLC), *Cucurbita pepo* + Cantaloupe + *L75* (CPCL75), *Cucurbita pepo* + Cantaloupe + *B. longum* (CPCLBF), *Cucurbita moschata* + Honeydew Control (CMHDC), *Cucurbita moschata* + Honeydew + *L75* (CMHD75), Cucurbita moschata + Honeydew + *B. longum* (CMHDBF), *Cucurbita pepo* + Honeydew Control (CPHDC), *Cucurbita pepo* + Honeydew + *L75* (CMHD75), and *Cucurbita pepo* + Honeydew + *B. longum* (CPHDBF).

**Table 2:** Changes in total phenolic compounds, total carotenoids, and different components of carotenoids in pumpkin leaf and melon smoothies fermented by different lactobacillus strains.

*Cucurbita pepo* and Watermelon Fermented Smoothie					
	Total Carotenoids (mg/100 mL)	Lutein (mg/100 mL)	Trans-β-Carotene (mg/100 mL)	Cis-β-Carotene (mg/100 mL)	Zeaxanthin (μg/mL) (mg/100 mL)
CPWC	3.27 ± 0.11 ^k,^*	1.55 ± 0.01 ^l^	31.51 ± 0.02 ^l^	1.02 ± 0.04 ^k^	0.06 ± 0.05 ^g^
CPW75	7.38 ± 0.02 ^g^ (2.26-fold) **	1.63 ± 0.05 ^j^ (1.05-fold)	102.64 ± 0.03 ^i^ (3.26-fold)	7.35 ± 0.1 ^g^ (7.21-fold)	0.19 ± 0.14 ^f^ (3.16-fold)
CPWBF	7.31 ± 0.12 ^g^ (2.24-fold)	1.83 ± 0.00 ^h^ (1.19-fold)	98.29 ± 0.05 ^j^ (3.11-fold)	2.10 ± 0.23 ^i^ (2.05-fold)	0.17 ± 0.00 ^f^ (2.83-fold)
*Cucurbita moschata* and Watermelon fermented smoothie					
CMWC	4.12 ± 0.0 ^j^	1.57 ± 0.00 ^k^	47.39 ± 0.08 ^k^	1.48 ± 0.32 ^j^	0.08 ± 0.01 ^g^
CMW75	5.01 ± 0.16 ^h^ (1.22-fold)	1.69 ± 0.00 ^i^ (1.07-fold)	358.60 ± 0.07 ^g^ (7.57-fold)	7.35 ± 0.42 ^g^ (4.97-fold)	0.36 ± 0.05 ^e^ (4.50-fold)
CMWBF	4.77 ± 0.02 ^i^ (1.16-fold)	2.30 ± 0.00 ^c^ (1.46-fold)	125.45 ± 0.04 ^h^ (2.65-fold)	3.40 ± 0.31 ^h^ (2.30-fold)	0.32 ± 0.01 ^e^ (4.0-fold)
*Cucurbita pepo* and Cantaloupe fermented smoothie					
CPCLC	14.16 ± 0.07 ^e^	2.07 ± 0.00 ^g^	396.09 ± 0.00 ^f^	11.69 ± 0.06 ^f^	0.41 ± 0.03 ^d^
CPCL75	26.14 ± 0.17 ^a^ (1.85-fold)	2.53 ± 0.00 ^a^ (1.25-fold)	620.37 ± 0.00 ^a^ (1.57-fold)	28.20 ± 0.12 ^a^ (2.41-fold)	0.65 ± 0.02 ^b^ (1.58-fold)
CPCLBF	23.18 ± 0.00 ^b^ (1.64-fold)	2.25 ± 0.00 ^d^ (1.09-fold)	401.57 ± 0.00 ^d^ (1.01-fold)	15.40 ± 0.10 ^c^ (1.32-fold)	0.55 ± 0.01 ^c^ (1.34-fold)
*Cucurbita Moschata* and Cantaloupe fermented smoothie					
CMCLC	12.59 ± 0.02 ^f^	2.09 ± 0.00 ^f^	400.23 ± 0.00 ^e^	12.70 ± 0.04 ^e^	0.43 ± 0.02 ^d^
CMCL75	17.70 ± 0.03 ^c^ (1.41-fold)	2.51 ± 0.10 ^b^ (1.19-fold)	454.45 ± 0.00 ^b^ (1.14-fold)	25.43 ± 0.17 ^b^ (2.00-fold)	0.70 ± 0.03 ^a^ (1.62-fold)
CMCLBF	16.98 ± 0.02 ^c^ (1.35-fold)	2.20 ± 0.20 ^e^ (1.04-fold)	443.11 ± 0.00 ^c^ (1.11-fold)	13.42 ± 0.09 ^d^ (1.06-fold)	0.55 ± 0.03 ^c^ (1.27-fold)

Mean values were calculated based on three replicate samples. Different letters in the same column refer to statistical difference at *p*  <  0.05 according to Fisher’s protected LSD test. * Standard deviation, ** fold increase or decrease.

**Table 3:** Changes in antioxidant activities of pumpkin leaf and melon smoothies fermented by different Lactobacillus strains.

*Cucurbita pepo* Leaf and Watermelon Fermented Smoothie			
	FRAP (mM TEAC/mL)	DPPH (IC_50 µ_L/mL)	ABTS (IC_50 µ_L/mL)
CPWC (Control)	6.4 ± 0.058 ^i,*^	4.00 ± 0.03 ^j^	1.72 ± 0.01 ^h^
CPW75	7.00 ± 0.00 ^f^	3.47 ± 0.02 ^ef^	0.52 ± 0.03 ^f^
CPWBF	6.92 ± 0.10 ^g^	3.37 ± 0.01 ^e^	0.72 ± 0.06 ^f^
Cucurbita moschata leaf and Watermelon fermented smoothie			
	FRAP (mM TEAC/mL)	DPPH (IC50 µL/mL)	ABTS (IC50 µL/mL)
CMW (Control)	6.57 ± 0.20 ^i^	3.94 ± 0.06 ^i^	1.69 ± 0.00 ^h^
CMW75	9.89 ± 0.48 ^a^	3.15 ± 0.03 ^d^	0.45 ± 0.12 ^d^
CMWBF	7.12 ± 0.19 ^e^	3.17 ± 0.02 ^d^	0.83 ± 0.29 ^g^
Cucurbita pepo leaf and Cantaloupe melon fermented smoothie			
	FRAP (mM TEAC/mL)	DPPH (IC50 µL/mL)	ABTS (IC50 µL/mL)
CPCLC (Control)	6.16 ± 0.10 ^j^	3.86 ± 0.22 ^gh^	1.84 ± 0.12 ^i^
CPCL75	7.76 ± 0.04 ^b^	1.18 ± 0.07 ^a^	0.36 ± 0.04 ^c^
CPCLBF	7.27 ± 0.185 ^d^	2.80 ± 0.04 ^c^	0.39 ± 0.04 ^c^
Cucurbita moschata leaf and Cantaloupe melon fermented smoothie			
	FRAP (mM TEAC/mL)	DPPH (IC50 µL/mL)	ABTS (IC50 µL/mL)
CMCLC (Control)	6.14 ± 0.11 ^j^	3.77 ± 0.01 ^g^	1.84 ± 0.06 ^i^
CMCL75	7.40 ± 0.34 ^c^	2.66 ± 0.02 ^b^	0.17 ± 0.04 ^a^
CMCLBF	6.67 ± 0.05 ^h^	2.84 ± 0.04 ^c^	0.23 ± 0.44 ^b^

Mean values were calculated based on three replicate samples. Different letters in the same column refer to statistical difference at *p*  <  0.05 according to Fisher’s protected LSD test. * Standard deviation.

## Data Availability

The original contributions presented in the study are included in the article, further inquiries can be directed to the corresponding author.

## Supplementary Materials

The following supporting information can be downloaded at: https://www.mdpi.com/article/10.3390/foods13223562/s1, Table S1: Changes in color properties during fermentation with different lactobacillus strains on pumpkin leaf (*Cucurbita moschata* or *C. pepo)* and melon smoothies.
