# Supplementary material for: Impact of Fermentation of Pumpkin Leaves and Melon Varieties with Lactobacillus Strains on Physicochemical Properties, Antioxidant Activity, and Carotenoid Compounds
Source: Foods. 2024 Nov 7;13(22):3562. doi: 10.3390/foods13223562 (PMC11592831; doi:10.3390/foods13223562)
Supplement: Supplementary file 1 [file foods-13-03562-s001.zip › foods-3266105-supplementary.pdf]

Impact of Fermentation of Pumpkin Leaves and Melon Varieties with Lactobacillus Strains on Physicochemical Properties, Antioxidant activity, and Carotenoid Compounds

Pretty Mhlanga [1], Sephora Mianda & Dharini Sivakumar [1,2]

[1] Department of Crop Sciences, Tshwane University of Technology, Pretoria, 0001, South Africa; prettymhlanga99@gmail.com; miandamutombos@tut.ac.za; SivakumarD@tut.ac.za
[2] Centre for Nutrition & Food Sciences, Queensland Alliance for Agriculture and Food Innovation, The University of Queensland, QLD 4108, Queensland, Australia
Affiliation 2; d.sivakumar@uq.edu.au

* Correspondence: SivakumarD@tut.ac.za DS

Supplementary Table 1. Changes in colour properties during fermentation with different lactobacillus strains on pumpkin leaf (*Cucurbita moschata or C. pepo)* and melon smoothies

Treatments	Time (h)	a*	b*	L*	ΔE
		Cucurbita moschata + Watermelon			
CMWC	0	6.610±0.244 a*	9.623±0.231 a	23.63±0.511 c	
CMWC	2	5.383±0.059 b	8.657±0.025 b	20.73±1.163 e	3.449±0.677 gh
CMWC	24	4.290±0.050 e	8.413±0.120 bc	18.55±0.334 f	8.767±0.285 d
CMWC	48	4.050±0.027 e	5.643±0.367 g	17.16±0.070 g	10.871±0.510 b
CMWC	72	2.190±0.308 j	4.893±0.223 h	16.67±0.473 g	14.564±0.098 a
		Cucurbita moschata + Watermelon + *L75*			
CMW7	0	4.597±0.025 d	8.413±0.120 bc	22.36±0.339 d	
CMW75	2	3.610±0.069 f	8.063±0.362 c	21.66±0.036 d	1.260±0.390 jk
CMW75	24	2.630±0.036 hi	7.280±0.050 d	20.06±0.010 e	1.581±0.337 j
CMW75	48	2.467±0.114 i	6.180±0.451 f	18.73±0.120 f	3.935±0.407 g

CMW75	72	1.583±0.224 k	4.850±0.217 h	18.18±0.031 f	5.747±0.379 f
Cucurbita moschata + Watermelon + *B. longum*					
CMWB	0	4.873±0.064 c	6.723±0.031 e	29.14±0.720 a	
CMWBF	2	4.740±0.027 cd	6.387±0.074 ef	25.71±0.061 b	3.294±0.661 hi
CMWBF	24	3.617±0.085 f	6.047±0.065 f	20.49±0.234 e	5.714±0.488 f
CMWBF	48	3.270±0.269 g	5.573±0.006 g	18.45±0.509 f	8.016±0.295 de
CMWBF	72	2.730±0.154 h	5.160±0.035 h	14.82±0.090 h	9.505±0.636 c
Cucurbita pepo + Watermelon					
CPWC	0	3.723±0.031 b	9.323±0.163 a	23.57±1.793 a	
CPWC	2	3.680±0.017 bc	8.910±0.052 b	22.90±0.270 a	0.788±0.155 i
CPWC	24	3.105±0.064 f	8.453±0.031 c	16.51±1.096 de	7.141±0.943 c
CPWC	48	1.913±0.059 j	6.013±0.202 ef	16.27±0.036 ef	8.217±0.136 b
CPWC	72	1.730±0.112 k	5.327±0.467 h	16.12±0.257 ef	8.215±1.568 b
Cucurbita pepo + Watermelon					
CPW75	0	4.597±0.025 d	8.413±0.120 bc	22.36±0.339 d	
CPW75	2	3.610±0.069 f	8.063±0.362 c	21.66±0.036 d	1.260±0.390 h
CPW75	24	2.630±0.036 hi	7.280±0.050 d	20.06±0.010 e	1.892±0.337 h
CPW75	48	2.467±0.114 i	6.180±0.451 f	18.73±0.120 f	4.764±0.407 ef
CPW75	72	1.583±0.224 k	4.850±0.217 h	18.18±0.031 f	6.265±0.379 cd
Cucurbita pepo + Watermelon + *B. longum*					
CPWB)	0	4.873±0.064 c	6.723±0.031 e	29.14±0.720 a	
CPWBF	2	4.740±0.027 cd	6.387±0.074 ef	25.71±0.061 b	3.293±0.661 g
CPWBF	24	3.617±0.085 f	6.047±0.065 f	20.49±0.234 e	5.315±0.488 e
CPWBF	48	3.270±0.269 g	5.573±0.006 g	18.45±0.509 f	8.016±0.295 b
CPWBF	72	2.730±0.154 h	5.160±0.035 h	14.82±0.090 h	9.505±0.636 a
Cucurbita moschata + Cantaloupe					
CMCLC	0	-3.997±0.155 h	10.273±0.012 b	16.09±0.050 h	
CMCLC	2	-2.993±0.121 g	9.277±0.047 c	17.27±0.250 fg	7.385±0.206 d
CMCLC	24	-1.890±0.165 e	9.047±0.025 d	24.73±0.183 c	7.854±0.134 d
CMCLC	48	-1.370±0.035 bc	8.670±0.125 e	12.75±0.835 i	8.979±0.802 c
CMCLC	72	-0.653±0.127 a	7.710±0.254 g	12.10±0.533 i	10.056±0.541 b

		Cucurbita moschata + Cantaloupe + *L75*			
CMCL75	0	-4.030±0.099 h	10.273±0.012 b	16.62±0.859 gh	
CMCL75	2	-2.700±0.132 f	8.587±0.072 e	18.27±0.044 f	1.842±0.818 i
CMCL75	24	-1.847±0.006 de	7.880±0.040 f	26.14±0.105 b	4.542±0.755 g
CMCL75	48	-1.157±0.202 b	5.957±0.012 i	22.52±1.733 d	5.803±0.880 f
CMCL75	72	-1.157±0.208 b	4.513±0.080 k	20.24±0.006 e	8.9773±0.863 c
		Cucurbita moschata + Cantaloupe + *B. longum*			
CMCLBF	0	-4.197±0.051 h	11.123±0.006 a	17.23±0.320 fg	
CMCLBF	2	-2.737±0.012 f	9.363±0.045 c	19.73±0.172 e	3.388±0.973 h
CMCLBF	24	-1.603±0.131 cd	8.027±0.064 f	28.01±0.754 a	5.971±0.445 f
CMCLBF	48	-1.277±0.119 b	6.370±0.185 h	19.36±0.363 e	6.543±0.196 e
CMCLBF	72	-1.220±0.303 b	5.443±0.040 j	16.55±0.569 gh	11.512±0.356 a
		Cucurbita pepo + Cantaloupe			
CPCLC	0	-2.817±0.540 f	10.61±0.384 cd	13.66±0.142 i	
CPCLC	2	-2.537±0.315 ef	10.52±0.376 e	14.30±0.045 h	3.410±0.245 f
CPCLC	24	-1.800±0.210 cd	9.72±0.064 f	24.48±0.352 f	4.371±0.505 e
CPCLC	48	-1.113±0.280 ab	8.83±0.017 f	19.18±0.026 b	9.118±0.464 b
CPCLC	72	-1.040±0.399 ab	8.60±0.246 d	16.63±0.238 e	10.904±0.686 a
		Cucurbita pepo + Cantaloupe + *L75*			
CPCL7)	0	-2.483±0.045 ef	10.83±0.080 cd	13.67±0.165 hi	
CPCL75	2	-1.990±0.010 d	8.49±0.133 f	14.30±0.045 g	0.704±0.136 g
CPCL75	24	-1.463±0.121 bc	6.61±0.238 h	21.34±0.886 a	1.314±0.742 h
CPCL75	48	-1.147±0.155 ab	8.37±0.035 f	15.42±0.351 g	3.301±0.221 f
CPCL75	72	-1.040±0.399 ab	10.52±0.376 d	14.18±0.026 hi	8.848±0.482 c
		Cucurbita pepo + Cantaloupe + *B. longum*			
CPCLB)	0	-5.147±0.549 h	16.44±0.442 a	20.98±0.354 d	
CPCLBF	2	-3.387±0.070 g	14.41±0.366 b	23.08±0.570 c	1.562±0.530 g
CPCLBF	24	-2.143±0.291 de	11.04±0.507 c	23.47±0.172 c	4.002±0.322 e
CPCLBF	48	-0.920±0.035 a	8.59±0.245 f	19.07±0.616 e	6.045±0.610 d
CPCLBF	72	-0.830±0.139 a	7.47±0.182 g	18.57±0.338 e	10.24±0.290 a
		Cucurbita moschata + Honeydew			

CMHDC	0	-3.140±0.027 i	9.230±0.061 a	14.20±0.000 k	
CMHDC	2	-2.750±0.017 h	8.853±0.015 b	15.61±0.032 h	1.511±0.057 g
CMHDC	24	-1.310±0.010 e	8.843±0.057 b	16.42±0.120 f	2.903±0.121 e
CMHDC	48	-1.115±0.050 bc	7.043±0.201 i	10.48±0.051 m	4.766±0.151 c
CMHDC	72	-1.057±0.031 ab	6.957±0.021 j	9.64±0.006 n	6.648±0.041 a
Cucurbita moschata + Honeydew + *L75*					
CMHD7)	0	-2.840±0.020 h	9.173±0.050 a	17.77±0.015 d	
CMHD75	2	-2.807±0.042 h	8.610±0.000 c	19.22±0.042 c	1.556±0.061 g
CMHD75	24	-1.500±0.010 f	7.687±0.055 f	24.11±0.000 a	2.774±0.019 e
CMHD75	48	-1.213±0.047 d	7.327±0.021 h	16.49±0.038 f	3.726±0.046 d
CMHD75	72	-1.047±0.040 ab	6.833±0.029 k	15.49±0.053 i	5.504±0.048 b
Cucurbita moschata + Honeydew + *B. longum*					
CMHDBF	0	-2.817±0.104 h	8.363±0.038 d	15.96±0.026 g	
CMHDBF	2	-2.270±0.020 g	7.833±0.074 e	16.77±0.012 e	1.112±0.085 h
CMHDBF	24	-1.153±0.047 cd	7.870±0.000 e	20.21±0.007 b	2.191±0.095 ef
CMHDBF	48	-1.350±0.036 e	7.550±0.000 g	14.55±0.000 j	3.522±0.104 d
CMHDBF	72	-1.000±0.132 a	7.337±0.045 h	12.95±0.021 l	4.591±0.041 c
Cucurbita pepo + Honeydew					
CPHDC	0	-2.937±0.123 ef	10.427±0.055 c	20.13±0.261 g	
CPHDC	2	-2.597±0.064 d	10.183±0.038 d	20.60±0.040 f	3.299±0.225 f
CPHDC	24	-2.653±0.517 de	9.720±0.036 e	23.34±0.319 d	5.087±0.214 cd
CPHDC	48	-1.257±0.015 bc	8.460±0.079 g	16.78±0.095 i	7.070±0.178 b
CPHDC	72	-1.093±0.060 ab	7.647±0.015 jk	13.38±0.156 k	11.690±0.111 a
Cucurbita pepo + Honeydew +*L75*					
CPHD7)	0	-2.967±0.023 f	12.650±0.027 a	22.91±0.644 e	
CPHD75	2	-2.840±0.027 def	11.400±0.070 b	25.77±0.405 b	0.629±0.243 h
CPHD75	24	-2.620±0.193 d	10.443±0.146 c	27.48±0.040 a	3.124±0.639 fg
CPHD75	48	-1.493±0.102 c	8.070±0.040 h	17.73±0.036 h	4.232±0.613 e
CPHD75	72	-1.343±0.031 bc	7.690±0.072 j	12.45±0.116 l	7.529±0.530 b
Cucurbita pepo + Honeydew +*L75*					
CPHDBF	0	-2.853±0.006 def	8.900±0.085 f	20.76±0.214 f	

CPHDBF	2	-2.783±0.021 def	8.167±0.025 h	23.90±0.145 c	3.225±0.092 f
CPHDBF	24	-2.613±0.331 d	7.843±0.082 i	25.73±0.000 b	3.816±0.335 ef
CPHDBF	48	-1.213±0.032 bc	7.550±0.010 kl	17.59±0.000 h	5.087±0.221 cd
CPHDBF	72	-0.910±0.070 a	7.450±0.036 l	15.42±0.173 j	5.865±0.053 c

Mean values were calculated based on three replicate samples. Different letters in the same column refer to statistical difference at $p < 0.05$ according to Fisher's protected LSD test. *Standard deviation

Keys: Cucurbita moschata + Watermelon (CMWC), *Cucurbita moschata* + Watermelon + *L75* (CMW75), *Cucurbita moschata* + Watermelon + *B. longum* (CMWBF), *Cucurbita pepo* + Watermelon Control (CPWC), *Cucurbita pepo* + Watermelon + *L75* (CPW75), *Cucurbita pepo* + Watermelon + *B. longum* (CPWBF), *Cucurbita moschata* + Cantaloupe Control (CMCLC), *Cucurbita moschata* + Cantaloupe + *L75* (CMCL75), *Cucurbita moschata* + Cantaloupe + *L75* (CMCL75), *Cucurbita pepo* + Cantaloupe Control (CPCLC), *Cucurbita pepo* + Cantaloupe + *L75* (CPCL75), *Cucurbita pepo* + Cantaloupe + *B. longum* (CPCLBF), *Cucurbita moschata* + Honeydew Control (CMHDC), *Cucurbita moschata* + Honeydew + *L75* (CMHD75),Cucurbita moschata + Honeydew + *B. longum* (CMHDBF), *Cucurbita pepo* + Honeydew Control (CPHDC),*Cucurbita pepo* + Honeydew + *L75* (CMHD75) and *Cucurbita pepo* + Honeydew + *B. longum* (CPHDBF)

Analysed data for Antioxidant activity showing significant differences

Analysis of variance

Variate: ABTS

Source of variation	d.f.	s.s.	m.s.	v.r.	F pr.
TMT	11	20.831956	1.893814	206.43	<.001
Residual	24	0.220181	0.009174		
Total 35 21.052137					

Least significant differences of means (5% level)

Table	TMT
rep.	3
d.f.	24
l.s.d.	0.1614

Analysis of variance

Variate: DPPH

Source of variation	d.f.	s.s.	m.s.	v.r.	F pr.
TMT	11	21.009128	1.909921	1470.03	<.001
Residual	24	0.031182	0.001299		
Total	35	21.040309			

Least significant differences of means (5% level)

Table	TMT
rep.	3
d.f.	24
l.s.d.	0.06074

Analysis of variance

Variate: FRAP

Source of variation	d.f.	s.s.	m.s.	v.r.	F pr.
TMT	11	30.24204	2.74928	42.61	<.001
Residual	24	1.54842	0.06452		

Total 35 31.79046

Least significant differences of means (5% level)

Table	TMT
rep.	3
d.f.	24
l.s.d.	0.4280
